# iTRAQ-based proteomic profiling of *Vibrio parahaemolyticus* under various culture conditions

**DOI:** 10.1186/s12953-015-0075-4

**Published:** 2015-07-29

**Authors:** Wenxin Yang, Dewen Ding, Chundan Zhang, Jun Zhou, Xiurong Su

**Affiliations:** School of Marine Science, Ningbo University, Ningbo, 315211 P.R. China; College of Life Science, Liaoning Normal University, Dalian, 116081 P.R. China

**Keywords:** *Vibrio parahaemolyticus*, Quantitative proteomics, iTRAQ, Biomarker, Pathogenicity

## Abstract

**Background:**

*Vibrio parahaemolyticus* is a common pathogen infecting humans and marine animals; this pathogen has become a major concern of marine food products and trade. In this study, *V. parahaemolyticus* isolated from sewage was exposed to different culture conditions and analyzed by isobaric tag for relative and absolute quantitation (iTRAQ) based reversed-phase liquid chromatography-tandem mass spectrometry (LC-MS/MS) technique. Our goal is to gain further insights into the proteomics of *V. parahaemolyticus*, particularly differentially expressed proteins closely correlated with growth conditions and pathogenicity associated proteins.

**Results:**

In this study, a total of 2,717 proteins including numerous membrane proteins were significantly identified, and 616 proteins displayed significant differential expression under different conditions. Of them, 12 proteins mainly participating in metabolism showed the most elastic expression differentiation between different culture conditions. Some membrane proteins such as type I secretion outer membrane protein, TolC, lipoprotein, efflux system proteins iron-regulated protein A and putaive Fe-regulated protein B, ferric siderophore receptor homolog and several *V. parahaemolyticus* virulence-associated proteins were differentially regulated under different conditions. Some differentially regulated proteins were analyzed and confirmed at gene expression level by quantitative real time polymerase chain reaction (qRT-PCR).

**Conclusions:**

Proteomics analysis results revealed the characteristics of *V. parahaemolyticus* proteome expression, provided some promising biomarkers related with growth conditions, the results likely advance insights into the mechanism involved in the response of *V. parahaemolyticus* to different conditions. Some virulence-associated proteins were discovered to be differentially expressed under different conditions.

**Electronic supplementary material:**

The online version of this article (doi:10.1186/s12953-015-0075-4) contains supplementary material, which is available to authorized users.

## Introduction

*Vibrio parahaemolyticus* is a common opportunistic pathogen infecting humans and marine animals; this pathogen causes food-borne gastroenteritis, occasional wound infection, and sepsis in immune-compromised patients, as well as great losses in crustacean and fish aquaculture. *V. parahaemolyticus* has been considered as a significant public health concern and sanitary risk in the production and trade of seafood worldwide because this species is abundant in marine products [1]. Numerous cases of *V. parahaemolyticus* infection have been reported in East Asia, South East Asia, North America, and others [2–7]; as such, *V. parahaemolyticus* has been recognized as pandemic. In 2004, a highly virulent strain caused a major outbreak with more than 1,000 cases in Chile [8].

*V. parahaemolyticus* is widely distributed in estuarine, marine, and coastal environments [9]. Majid Alipour et al. [10] detected 62 (20.3 %) *V. parahaemolyticus* strains from 300 seawater and sediment samples in the southern coast of the Caspian Sea. Cabrera-Garcia et al. [11] reported that 15 % of the seawater samples in the Gulf of Mexico contained *V. parahaemolyticus*. We also isolated *V. parahaemolyticus* strains multiple times in sewage and adjacent seawaters of Dalian, China, in different seasons.

Almost all of the clinical *V. parahaemolyticus* isolates exhibit β-hemolysis on Wagatsuma agar, and this phenomenon is known as Kanagawa phenomenon, which is induced by thermostable direct hemolysin (TDH) produced by *V. parahaemolyticus* and has been considered a crucial marker that distinguishes pathogenic strains from non-pathogenic strains [12]. Since 1996, “pandemic clones” mainly belonging to sero type O3:K6, have caused gastroenteritis outbreaks in India and other parts of the world. More than 50 % of *V. parahaemolyticus* strains isolated from patients in India are of sero type O3:K6 [2, 8]. *V. parahaemolyticus* outbreak rapidly spread to other countries in Asia, South America, North America, Africa, and Europe, resulting in a pandemic that affected numerous individuals [13–16]. In 1998, a new highly virulent strain was responsible for a large gastroenteritis outbreak in Galveston Bay, Texas [1].

Although *V. parahaemolyticus* has been recognized for many years, the response of this species to different environments at a proteome level remains unclear. Proteomics aims to monitor global proteins in a cell or an organism, reveal plasticity in terms of development and environment, and evaluate gene expression, protein–protein interactions, and correlation between proteome expression and environment. Advances in proteomics based on tandem MS and applications of isobaric peptide and protein labeling for relative quantification provide promising tools to discover biomarkers and elucidate the molecular regulatory mechanisms underlying responses to different environments. In this study, *V. parahaemolyticus* was subjected to proteomic analysis by iTRAQ labeling to identify differentially expressed proteins upon exposure to different growth environments. iTRAQ is a powerful tool to relatively and absolutely quantify proteins, and has been extensively applied to proteome analysis since this technology was invented in 2004 [17–22].

To the best of our knowledge, this study is the first to perform a detailed proteomic analysis of *V. parahaemolyticus* in different growth environments.

## Results and discussion

### Protein identification

**Fig. 1:**
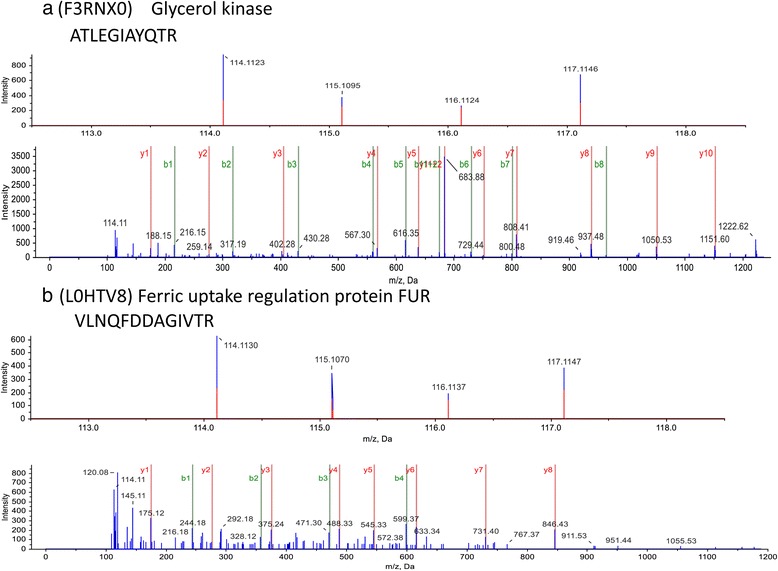
Representative MS/MS spectra of peptides from differentially expressed proteins. **a**. ATLEGIAYQTR peptide belongs to glycerol kinase. **b**. VLNQFDDAGIVTR peptide belongs to ferric uptake regulation protein FUR. The spectra indicate the relative intensity of reporter ions (VPP/114, VPE/115, VPX/116, and VPW/117) from MS/MS fragmentation

A total of 2,717 proteins or approximately 60 % of the 4,832 predicted Open Reading Frames in the genome (*V. parahaemolyticus*, RIMD2210633) were significantly identified from 70,197 MS/MS spectra and 28,006 peptides using 1 % false discovery rate (FDR) as cutoff in the triplicate independent experiments. The result showed a very wide coverage of the protein identification method utilized in this study. The MS/MS spectra of representative peptides belonging to two differentially expressed proteins are shown in Fig. 1.

**Fig. 2:**
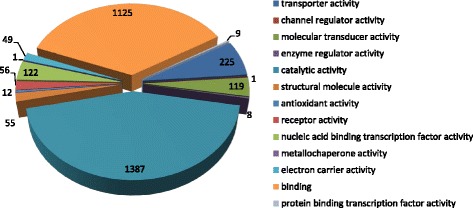
Gene ontology enrichment analysis of molecular function of the identified proteins. The classification of the molecular function of the identified proteins is based on a UniProt KB search and KEGG pathway analysis

Among 2,717 proteins, 2,110 were analyzed in term of their gene ontology (GO) annotations in UniProtKB database (http://www.uniprot.org/) [23]. Based on GO classification, their molecular functions were mainly displayed in catalytic activity with approximately 44 % of all molecular functions, binding function takes up 35.50 %, transporter activity takes up 7.10 %, and nucleic acid binding transcription factor activity takes up 3.85 % (Fig. 2).

**Fig. 3:**
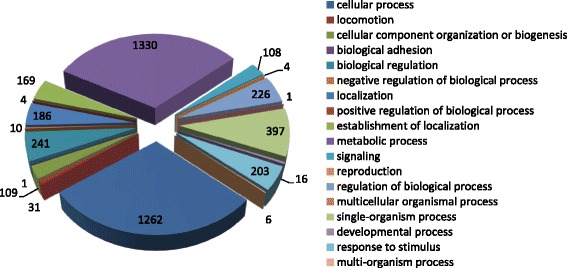
Gene ontology enrichment analysis of biological process of the identified proteins. The classification of the biological process of the identified proteins is based on a UniProt KB search and KEGG pathway analysis

Analysis of biological processes revealed that most proteins were involved in metabolic processes accounting for 30.90 % of all biological processes (1330/4303), 29.37 % of cellular processes, 5.25 % of regulation of biological processes, and 4.72 % of responses to stimuli (Fig. 3). In addition, more than 500 proteins (approximately 20 % of the proteins identified in this study) were not assigned annotated function.

In this study, numerous membrane proteins were significantly identified, they included 1 putative porins OmpU; 2 TolC family (outer membrane protein TolC, " type I secretion outer membrane protein TolC); 4 OmpA family proteins (outer membrane protein OmpA, OmpA family protein OmpA, outer membrane protein OmpA, outer membrane protein A); 4 Iron-regulated proteins (Iron-regulated outer membrane virulence protein homolog, iron-regulated protein A, putaive Fe-regulated protein B, Iron-regulated virulence regulatory protein homolog); 2 receptor proteins (cyclic AMP receptor protein, outer membrane protein OmpK); 13 transport proteins (heme transport protein HutA, Long-chain fatty acid transport protein, ferrous iron transport protein B, biopolymer transport protein ExbB-related protein, magnesium and cobalt transport protein CorA, Putative transport protein, etc.); 9 polysaccharide-related proteins (putative polysaccharide export-related protein, polysaccharide biosynthesis/export protein, putative polysaccharide export-related protein, etc.); 27 lipoprotein (lipoprotein, thiamin biosynthesis lipoprotein ApbE, apolipoprotein N-acyltransferase, etc.); 11efflux pump proteins (ABC- type multidrug efflux pump, Putative multidrug efflux membrane fusion protein, RND multidrug efflux transporter, putative Co Zn Cd efflux system membrane fusion protein, putative cation efflux system, glutathione-regulated potassium-efflux system protein KefB, etc.); 3 pilins(pilin protein MshA, type 4 prepilin-like proteins leader peptide-processing enzyme, pilin protein MshA).

We also discovered several types of secretion system proteins, they were 2 type I secretion (type I secretion outer membrane protein TolC, putative transport protein); 2 type II secretion (type II secretion system protein L, type II secretion system protein K); 7 type III secretion system (T3SS) protein (YscC, T3SS ATPase, T3SS cytoplasmic protein YscL, Putative translocation protein in T3SS, etc.) and 2 type VI secretion protein (type VI secretion ATPase, ClpV1 family and type VI secretion protein, VC_A0110 family) as well as 11 general secretion pathway proteins.

Other virulence related proteins such as three hemolysins(GN VP0730, VP2536, VPA0257) and regulator proteins Hfq and rsmA were also significantly identified.

### Protein quantification and differentiation analysis

Proteins with iTRAQ ratios <0.50 or >2.0, *p* < 0.05 were considered significantly different in protein quantification. Following these criteria, a total of 2,643 proteins, or more than 97 % of the identified proteins were accurately quantified by iTRAQ labeling coupled with RP–LC interfaced with Triple 5600 mass spectrometer (AB sciex) (iTRAQ ratios of the MS/MS spectra of representative peptides belonging to two differentially expressed proteins are illustrated in Fig. 1).

A total of 616 proteins were significantly regulated in VPP, VPE, and VPX compared with VPW. The numbers of the differentially expressed proteins under different conditions were listed in Table 1. An additional file showed the GO enrichment analysis of differentially regulated proteins in VPP, VPE, VPX, and VPW (see Additional file 1: Table S1 and S2).

**Table 1:** Number of the differentially expressed proteins of *V. parahaemolyticus* among different samples

Comparison	Number of differentially expressed proteins		
	Total number	Up-regulated	Down-regulated
VPP(114)/VPW(117)	97	47	50
VPE(115)/VPW(117)	281	147	134
VPX(116)/VPW(117)	400	184	216
VPE(115)/ VPP(114)	331	178	153
VPX(116)/ VPP(114)	448	197	251
VPE(115)/ VPX(116)	567	331	236

Only 97 proteins were differentially expressed in VPP compared with VPW, by contrast, 281 proteins were significantly altered in VPE compared with VPW and 400 proteins displayed significant differential abundance between VPX and VPW. The result indicated that *V. parahaemolyticu* presented highly variable protein expression and growing environment had a direct effect on protein expression of *V. parahaemolyticus*.

**Fig. 4:**
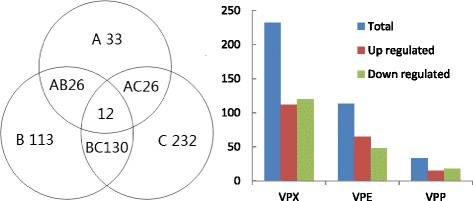
Numbers of unique differentially expressed proteins under different conditions. In the Venn diagram, A, B, and C indicate the number of significantly unique regulated proteins in VPP, VPE, and VPX compared with VPW, respectively; AB, AC, and BC indicate the overlap of the proteins simultaneously differentially expressed under the two conditions compared with VPW; 12 indicates the number of the proteins simultaneously differentially expressed in VPP, VPE, and VPX compared with VPW;. The column diagram indicates the number of up-regulated and down-regulated unique proteins in VPP, VPE, and VPX

Some protein was regulated only in some condition. Of the 616 significantly regulated proteins, 33 proteins were regulated only in VPP compared with VPW (15 were up-regulated, 18 were down-regulated); 113 were only found to altered VPE versus VPW (65 up-regulated, 48 down-regulated); and the abundance of 232 proteins was differently regulated in VPX (112 up-regulated, 120 down-regulated) (Fig. 4). The detailed information of the proteins was listed in Additional files 2, 3 and 4. These results further indicated that the detected *V. parahaemolyticus* required different factors to transport various ions, nutrients, and other metabolites across the outer membranes under different growth conditions; *V. parahaemolyticus* also required specific signal pathways that respond to various environmental stimuli. In another study, *V. splendidus* was reported to forms distinct populations in different ecological niches in marine environment [24]. Keymer et al. [25] also demonstrated that a non-homogeneous marine environment stimulates the formation of diverse populations of *V. cholerae* strains.

Among the differentially expressed proteins, 12 were differentially regulated in VPP, VPE, and VPX compared with VPW without exception (Table 2). The abundance of most of these proteins was increased in VPP, VPE, and VPX compared with VPW. Function analysis results showed that the proteins with catalytic activity, including glycerol kinase (GN glpK; key enzyme in the regulation of glycerol uptake and metabolism), isocitrate dehydrogenase (GN VIPARAQ4037_2971), NADP-dependent, putative tricarboxylic transport TctC (GN VP1651), putative acyltransferase (GN VP10329_09192), and diaminobutyrate-pyruvate transaminase, mainly participate in the metabolism of carbohydrates, lipids, and proteins. The 12 differently regulated proteins exhibited the most elastic expression adapting to different growth conditions, they are promising biomarkers to monitor environment.

**Table 2:** Differentially expressed proteins in VPP, VPE, and VPX compared with VPW

Accession	Protein name	Changes in the relative abundance of proteins						
		VPP /VPW^a^	*P* Value^b^	VPE/VPW	*P* Value	VPX/VPW	*P* Value	GO
F3RNX0	Glycerol kinase	2.109↑^c^	0.048	0.425	0.007	0.233	2.0E-04	glycerol catabolic process
E1DA96	Isocitrate dehydroge nase, ADP-dependent	2.399↑	0.026	2.377↑	1.38E-06	0.071	1.1E-06	tricarboxylic acid cycle
L0HV77	30S ribosomal protein S4	0.308	0.006	2.679↑	0.007	0.302	4.0 E-04	translation
F3RYI1	putative acyltransferase	2.858↑	0.001	0.154	0.007	2.884↑	7.61E-05	acetyl-CoA C-acetyltransferase activity
E1EMA1	Carbamoyl-phosphate synthase small chain	2.535↑	0.049	2.655↑	0.022	7.656↑	1.70E-06	glutamine catabolic process
L0I0Y7	Ribosomal protein S6 modification protein	0.092	5.71E-06	0.291	9.22E-05	0.461	0.002	cellular protein modification process
Q87SB9	ATP-dependent RNA helicase SrmB ATP	2.965↑	0.048	3.981↑	0.004	6.081↑	0.001	ATP-dependent helicaseactivity
Q87P98	Amino acid ABC transporter, periplasmic amino acid-binding protein	4.093↑	0.001	0.191	0.001	9.462↑	3.95E-06	transporter activity
F3RQ38	Diaminobutyrate-pyruvate transaminase and L-2,4-diaminobutyrate decarboxylase	0.217	0.021	2.535↑	0.001	0.146	0.014	carboxylic acid metabolic process
Q87P67	Putative tricarboxylic transport TctC	4.325↑	0.003	4.325↑	0.002	2.992↑	0.013	outer membrane-bound periplasmic space
L0HXG6	ABC- type antimicrobial peptide transport system, permease component	6.252↑	0.016	2.489↑	0.041	15.136↑	0.008	integral to membrane
Q87PV1	Uncharacterized protein	3.664↑	0.048	10.568↑	0.012	3.565↑	0.014	protein serine/threonine kinase activity

^a^iTRAQ ratio indicates the relative quantification of the differentially expressed proteins

^b^Statistical analysis of iTRAQ ratio was performed using unpaired *t*-test

^c^Up arrows indicate an increase in protein expression

Functional analysis of the proteins with different abundance showed the regulated proteins could be clustered into 4 major groups: 1) metabolic proteins; 2) proteins involved in transcription and translation; 3) membrane-associated proteins including proteins involved in transport, antibiotic efflux system, secretion system, outer membrane proteins, etc.; 4) virulence factors including proteins involved in iron acquisition, secreted protease, etc. In this paper, we would pay emphasis on analysis of the latter three groups.

A number of proteins related to DNA replication, cell division and transcriptional regulation were observed to be differentially regulated in some conditions. Most of proteins participated in chromosome structure, DNA replication, and transcription were down-regulated in VPP (Additional file 2: Table S3). However, 20 translation-related proteins were present at increased abundance in VPE, including peptide chain release factor 1, translation initiation factor IF-2, elongation factor Ts, elongation factor Tu, 30S ribosomal proteins (S9, S10, S8, S11, S13, S6, and S18), and 50S ribosomal proteins (L6,L9, L13, L31, and L19) (Additional file 3: Table S4). 30S small ribosomal subunit proteins and 50S large ribosomal subunit proteins constitute ribosome and exhibit different functions in protein synthesis.

Surface proteins can directly participate in microbial virulence by facilitating pathogen dissemination via interactions with host factors. For example, outer membrane proteins are located at host–bacterial interface and are important for host immune responses and as targets for drug therapy [26]. Drug efflux pumps can participate in drug resistance to multiple antimicrobials through export drugs, and also serve other functions in bacteria. Recently, antibiotic-resistant strains of the bacterium from clinical and environmental sources have been frequently reported [27, 28]. Investigation of secretion systems is often critical to understanding the virulence mechanisms of bacterial pathogens. It was estimated that as high as 30–40 % of proteins were secreted or localized to the cell envelope. So far, seven different secretion systems, type I-VII, have been described in bacteria [29, 30]. These secretion systems release factors that modulate the host environment to favor bacterial fitness and or virulence.

In this study, numerous membrane proteins were identified and some membrane proteins were differentially regulated in some condition. VPX displayed highly elevated abundance of type I secretion outer membrane protein TolC, lipoprotein, apolipoprotein N-acyltransferase, long-chain fatty acid transport protein, magnesium and cobalt transport protein CorA, but a putative polysaccharide export-related protein displayed reduced. Moreover, a number of efflux system proteins, including putative multidrug efflux membrane fusion protein, ABC transporter, periplasmic substrate-binding protein, phosphate ABC transporter, and permease protein PstA were present at increased abundance in VPX. And chemotaxis proteins, such as putative chemotaxis transducer (GN VPA0562), methyl-accepting chemotaxis proteins (GN VP1904, VIPARK5030_0929, VPBB_A0559, VPA0511), and magnesium and cobalt transport protein CorA were up-regulated similarly.

Many proteins involved in *V. parahaemolyticus* virulence were significantly up-regulated in VPX; this result could be attributed to the blood component of blood agar, which suggested that the pathogenicity of *V. parahaemolyticus* is likely enhanced in blood after infecting animals. Nevertheless, further studies should be conducted to elucidate the underlying mechanisms.

**Fig. 5:**
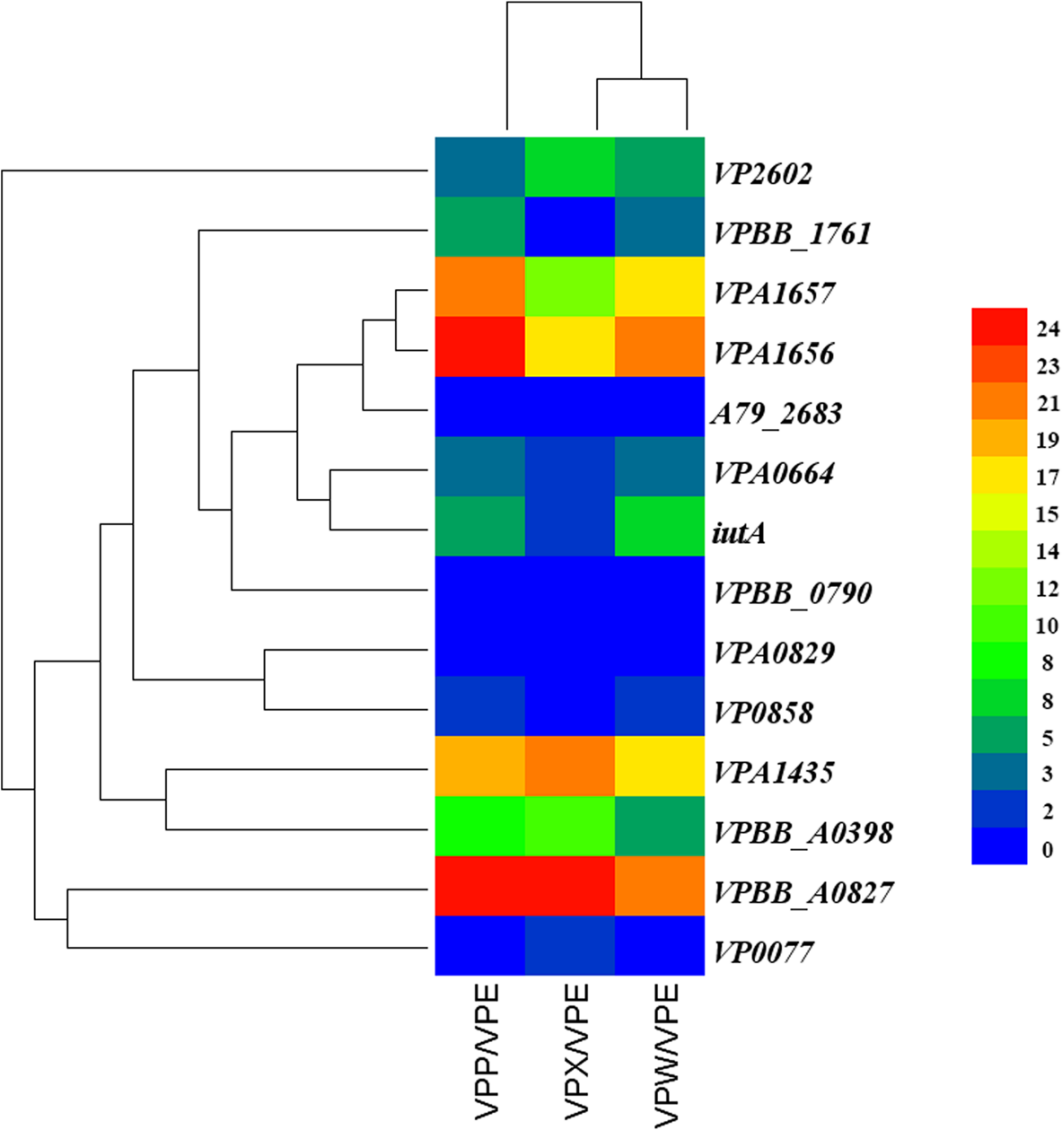
Differential expression of iron-responsive proteins in VPP, VPE, and VPW compared with VPE. Red indicates higher expression, blue indicates lower expression, and graphic symbols indicate the ratio change of expression between the strains. The proteins were grouped depending on their expression level

Iron availability was reported to play a major role in the virulence of bacterium [31]. We discovered that four iron-regulated proteins, iron-regulated outer membrane virulence protein homolog (GN VP2602), iron-regulated protein A (GN VPBB_1761), putative Fe-regulated protein B (GN VPA0664) and putative iron compound receptor (GN VPA1435) and four transport proteins including ferric siderophore receptor homolog (GN VPA1657), heme transport protein HutA (GN VPBB_A0827), ferric vibrioferrin receptor (GN VPA1656) and ferric aerobactin receptor(GN *iutA*) were significantly decreased in VPE compared with VPP, VPW and VPX, namely they were significantly increased relatively in VPP, VPW and VPX (Fig. 5). Pyridoxamine 5'-phosphate oxidase-related putative heme iron utilization protein (GN VPBB_A0398) also was down-regulated. Only iron-containing alcohol dehydrogenase (GN VPA0829) was significantly up-regulated. Other proteins involved in iron maintenance, including ferrous iron transport protein B (GN VP0858); ferric uptake regulatory protein, bacterioferritin (GN VP2768), co-migratory protein(GN A79_2683) and ferritin (GN VP0077) was increased to some extent but not to a significantly different degree.

Ferric uptake regulatory protein Fur (GN VPBB_0790) containing sequence-specific DNA binding transcription factor is the master regulator as a repressor of iron acquisition-related genes [32], Miyamoto *et a*l. [33] showed that the Fur protein regulates the expression of the virulence-associated orthologous groups in the family *Vibrionaceae*. In this study, increased Fur level was consistent with decreased abundances observed for several iron acquisition proteins in VPE (Fig. 5).

Iron acquisition via siderophore production is critical for successful colonization and for providing bacterium with a distinct competitive advantage over other pathogens [20]. In this study, protein expression of *V. parahaemolyticus* displayed a unique feature on the FeCl_3_ agar because of the ferric component. We discovered that the down-regulated proteins in VPE were involved in iron uptake, while up-regulated proteins were involved in iron storage. The results suggesting *V. parahaemolyticus* was adapted to an iron-rich environment, the species can maintain intracellular iron concentration at a relatively stable level by self-adjusting mechanisms. In other study, *Pseudomonas aeruginosa* was observed to be adapted to an iron-limited environment [20].

Several proteases, including the proteases of insulinase family protein, protease IV, protease HtpX, secreted microbial collagenase and putative membrane-associated Zn-dependent protease were evidently increased in VPX; however, ATP-dependent Clp protease were decreased. Protease is a virulence determinant [20], which suggested VPX may possess stronger virulence.

Information about the subcellular localization and organization of secretion system as well as identification and functional characterization of their substrates are key steps toward understanding these intricate systems. The role of type VI secretion system in virulence, symbiosis, biofilm formation, and stress response has been documented in several bacteria [34, 35]. In this study, type VI secretion protein-VC_A0110 family protein (GN VIPARK5030_1375) was up-regulated in VPE.

T3SS is possessed by gram-negative bacteria, especially those animal and plant pathogens, e.g., *Yersinia*, *Shigella*, *Salmonella*, *Pseudomonas* and *Escherichia* species [36, 37]. The T3SS secretes and translocates effector proteins into the cytosol of eukaryotic cells, thus contributing to bacterial virulence against the host [36], and *V. parahaemolyticus* was first reported to contain T3SS [38]. Previous study has showed that T3SS expression was effected by environmental factors [39], but the 7 T3SS proteins including Spa33, ATPase YscC and YscL, etc., were not significantly differentially expressed in this study.

Three hemolysin proteins including putative hemolysins (GN VP0730, GN VP2536) and hemolysin (GN VPA0257) were identified, although TDH production was reported to be affected by environmental factors, such as pH, temperature, and chemicals [40, 41], but significant differences in abundance have not been observed between the four culture conditions in this study. TDH and TDH-related hemolysin (TRH) are considered as the main virulence factors of *V. parahaemolyticus*, and pathogenetic *V. parahaemolyticus* strains can contain TDH, TRH, or both [12, 42, 43]. However, the presence of pathogenic populations of *V. parahaemolyticus* in environmental samples is generally low [3]. Presence of the *V. parahaemolyticus*strains containing TDH could be accounted for human fecal contamination in seawater from the sewage at the coast of Dalian. This information may be considered to prevent sanitary problems that may affect human health.

Two important regulator proteins Hfq and RsmA were successfully identified, they are two RNA binding proteins and major post-transcriptional regulators of gene expression. Increasing evidence shows that Hfq and its dependent sRNAs play a fundamental role in the regulation of stress response and pathogenesis [44]. Nakano et al. [45] investigated the effect of Hfq on the expression of virulence-associated genes including TDH using an *hfq* deletion mutant and observed that Hfq could depress the expression of TDH and may be involved in the pathogenicity of *V. parahaemolyticus*. RsmA (Ribosomal RNA small subunit methyltransferase A, GN *rsmA*) is the homologue of *E. coli* CsrA, was reported to play an important role in regulation of virulence and biocontrol factor production in *Pseudomonas aeruginosa* and *P. fluorescens* [46], and was examined to affect expression of some functional proteins including efflux transporters, outer membrane proteins, sigma factors, and stress response proteins in in *Serratia sp*. ATCC 39006 [17]. In this study, the two proteins were not significantly differentially expressed, however, Hfq was elevated to 1.318–1.542 folds in VPP, VPE and VPX compared with VPW, while the putative hemolysin was reduced to 0.597–0.614 fold, which was consistent with Nakano’s discovery.

### Correlation of expression between proteins and genes

**Fig. 6:**
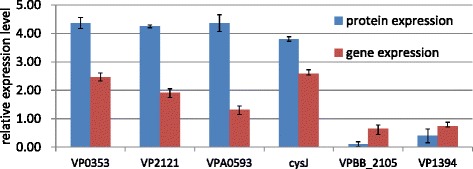
Comparison of iTRAQ result and qRT-PCR analysis. iTRAQ ratios of regulated proteins in VPX compared with VPW were confirmed by qRT-PCR analyses. The comparison of the fold changes showed the directions of protein regulation and gene regulation were the same for the 6 representative proteins. The former 4 proteins were up-regulated at both protein level and RNA level, and the latter 2 proteins were down-regulated. Blue bars values represent average protein expression ± SD and red bars values represent average gene expression ± SD from three independent experiments

In order to analyze the correlation between protein expression and gene expression and to confirm the accuracy of iTRAQ ratio obtained from the present proteomics study, we analyzed genes expression of 50 differentially expressed proteins in VPX compared with VPW by qRT-PCR. Data from triplicate experiments were analyzed for statistical significance by the unpaired *t*-test (two side test), with p < 0.05 being considered statistically significant. When the fold change of gene expression > 1.2 and P <0.05 indicated that the genes were regarded as regulated. Part results were showed as fold means ± standard deviation (SD) in Fig. 6.

Comparison of the fold changes showed the direction of change was the same for 39 proteins, about 80 % of detected proteins, at the level of both RNA and protein, which gave a positive Pearson correlation (Additional file 5: Table S6), and 9 proteins showed no significant correlation between the protein production and the mRNA transcript. However, 2 proteins, magnesium and cobalt transport protein CorA and alcohol dehydrogenase, iron-dependent presented negative correlation, which suggested a post-transcriptional effect on regulation. These results indicated that the iTRAQ ratios in present study are almost consistent with quantitative results obtained by qRT-PCR.

## Conclusions

This study is the first to perform quantitative proteomic investigation by iTRAQ labeling and LC-MS/MS to identify differentially expressed proteins in *V. parahaemolyticus* under different conditions. The results confirmed that *V. parahaemolyticus* presented a unique protein profile that indicated the adaptive mechanisms of this species to different environments. This profile could also provide promissing candidate proteins to detect environmental changes by using *V. parahaemolyticus*. Numerous membrane proteins such as type I secretion outer membrane protein TolC, lipoprotein, efflux system proteins and virulence associated proteins were characterized, some of them were significantly regulated. This study would advance our understanding of the evolution and pathogenicity of this food-borne pathogen.

## Materials and methods

### Source, isolation and culture of *V. parahaemolyticus*

*V. parahaemolyticus* strain was isolated from a sewage outlet in Dalian, China and selectively identified using thiosulfate-citrate-bile-sucrose agar medium (Haibo, China). The identity of *V. parahaemolyticus* was confirmed by 16S rRNA gene sequence analysis and analytical profile index tests. The identified and characterized pure culture was streaked onto sterile sewage agar (0.5 % beef extract, 1 % tryptone, 2 % agar, sterilized sewage) and seawater agar (0.5 % beef extract, 1 % tryptone, 2 % agar, sterilized seawater), the former mimics bacterial growth in sewage water and the latter mimics growth in seawater environment. In view of the hemolysis function of *V. parahaemolyticus* and requirement of ferric ion in metabolism, blood agar (0.5 % beef extract, 1 % tryptone, 2 % agar, 2 % sodium chloride, 5 % anticoagulant goat blood) and FeCl_3_ agar (0.5 % beef extract, 1 % tryptone, 2 % agar, 1 mmol/L FeCl_3_, sterilized seawater) were chosen to culture bacterium. Bacterium was cultured on every medium in triplicate. After the cultures were incubated at 37 °C for 12 h, the colonies on the four media were collected and named as VPW, VPP, VPE, and VPX matching sewage agar, seawater agar, FeCl_3_ agar and blood agar, respectively. The bacterial cultures were maintained at −80 °C for further analysis.

### Quantitative proteomics by iTRAQ and LC-MS/MS

#### Total protein extraction and quantification

Total protein was extracted from the colonies as described previously [20]; protein concentration was determined with Bradford colorimetric method (Bio-Rad) according to standard protocol. At least 100 μg of protein from each sample was lyophilized using a speed vacuum system (Martin Christ, Germany).

### iTRAQ labeling and strong cation exchange fractionation

iTRAQ labeling involves denaturation, reduction, trypsin digestion, and labeling. Protein samples were reduced using a reducing reagent (AB Sciex, PN: 4381664) for 1 h at 60 °C according to iTRAQ protocol. Cysteine was blocked using cysteine blocking reagent (AB Sciex, PN: 4381664) for 10 min at room temperature and then digested with trypsin (AB Sciex, PN: 4370285) overnight at 37 °C. iTRAQ (AB Sciex,PN:4381664) labeling of peptides was conducted according to the manufacturer’s protocol. In brief, peptides from *V. parahaemolyticus* colonies VPP, VPE, VPX, and VPW were labeled with iTRAQ reagents containing the reporters 114, 115, 116, and 117, respectively. Every sample was labeled in triplicate. Labeling was carried out for 2 h at room temperature. After labeling was performed, the peptides from the four colonies were pooled and fractionated by strong cation exchange chromatography on Durashell-C18 (4.6 mm × 250 mm, 5 μm 100 Å; Agela, Catalog Number: DC952505-0) to remove the remaining iTRAQ reagent and other reagents. The peptides were separated and eluted using a linear gradient of 0 to 70 % ACN for 70 min at a flow rate of 0.8 mL/min. Eluted peptides were monitored using a diode array detector at a wave length range of 200 nm to 400 nm. The eluted peptides were then collected at an interval of 1 min beginning at the first 5 min; a total of 48 fractionated components were collected per sample. The components were reduced to dryness by using a speed vacuum centrifuge.

### Mass spectrometry data analysis and protein quantification

Mass spectrometry analysis was performed using a reversed-phase liquid chromatography system (Eksigent) interfaced with Triple TOF™ 5600mass spectrometer (AB Sciex). MS/MS data were acquired by online analysis of peptides eluted using 5 to 80 % acetonitrile in 0.1 % formic acid for 100 min with a flow rate of 300 nL/min. MS/MS spectra were obtained in a data-dependent manner from *m*/*z* 350 to 1250 for TOF MS scan and from *m*/*z* 100 to1500 for product ion scan; these scans targeted the 10 most abundant ions in each survey scan with an accumulation period of 0.1 s and a dynamic exclusion period of 25 s.

MS data were analyzed by ProteinPilot 4.5(AB SCIEX, Foster City), which includes Paragon algorithm for identifying peptides and Pro Group algorithm for summarizing proteins. Search parameters were set as iTRAQ labeling at N-terminus and lysine residues, cysteine modification by Methylmethanethiosulfonate as fixed modifications, and trypsin as a protease. Proteins identified with 1 % false discovery rate (FDR) as determined by Pro Group algorithm were used for further analysis. The MS/MS data of 48 SCX fractions generated by LC–MS/MS analysis were searched against a database of predicted proteins reported for *V. parahaemolyticus*by using ProteinPilot 4.5 software. FDR was analyzed using PSPEP software in ProteinPilot 4.5. A threshold of 1 % FDR was used to identify and quantify proteins.

Proteins with a ratio <0.50 or >2.0were considered to be differentially expressed, and data from triplicate experiments were analyzed for statistical significance by unpaired t- test. In all cases, *p* < 0.05 was considered significant, namely, iTRAQ ratios <0.50 or >2.0 with *p* < 0.05 were considered significantly different in protein quantification.

### Gene ontology analysis of proteins and pathogenicity analysis

The identified proteins were subjected to GO analysis by using the analytical system of Generic Gene Ontology Term Finder (http://go.princeton.edu/cgi-bin/GO Term Finder) [47]. The fundamental functions of proteins were counted and analyzed; differentially expressed proteins were enriched into different functions with *p* ≤0.05 as threshold in the chi square test. The biological pathways of the proteins were acquired from the KEGG Pathway database (http://www.genome.ad.jp/kegg/) [48] coupled with UniProtKB annotation (UniProtKB database, http://www.uniprot.org/) [23]. The abundance of virulence-related proteins was analyzed to reveal the effect of growth conditions on the pathogenicity of *V. parahaemolyticus* strain*.*

### qRT-PCR analysis

#### Total RNA extraction

Total RNA was extracted from VPE and VPW when *V. parahaemolyticus* was cultured for 12 h at 37 °C. RNA was extracted using the hot phenol method as previously described [49]. Precipitated RNA was re-suspended in 100 μL H_2_O and treated with DNase I (Roche Diagnostics, Basel, Switzerland), according to the manufacturer’s protocol. The RNA was incubated at 37 °C for 30 min. The concentration of the RNA was verified by absorbance measurements at 260 nm and the purity was verified by its A260/280 ratio with a Nanodrop ND-1000 spectrophotometer (NanoDrop Technologies Inc, USA), the integrity was verified by electrophoresis separation using the RNA Nano kit (Agilent Technologies).

### qRT-PCR

Regulation of the gene expression matching 50 regulated proteins in VPX compared with VPW was analyzed by qRT-PCR. 4 μg of total RNA was transcribed to cDNA using oligodT primers and SuperScript II Reverse Transcriptase Kit as recommended (Invitrogen). qRT-PCR reaction was performed in triplicate using the ABI Prism 7900HT sequence detection system (Applied Biosystems) with SYBR^®^ Premix Ex TaqTM II according to the manufacturer’s protocols. Oligonucleotide primers for quantitative PCR are listed in Additional file 5: Table S6. Reaction mixture contained 10 μL SYBR^®^ Premix Ex TaqTM II 2×, 1 μL (10 μM) of reverse and forward primer, and 2.5 μL of cDNA in a final volume of 25 μL. The thermal amplification was performed as follow: initial denaturation at 95 °C for 1 min, followed by 40 cycles with 30 s at 95 °C, 15 s annealing at 55 °C and 30s at 72 °C, followed by a single fluorescence measurement. Relative gene expression was obtained using 16S rRNA as the control with mRNA/16S rRNA = 1 in the VPW. Gene expression data obtained from PCR reaction were evaluated using Q-Gene.

## Additional files

Additional file 1: Table S1. GO enrichment analysis of proteins with differential expressions. **Table S2.** GO enrichment analysis of proteins with differential expressions (biological process). (XLSX 14 kb) Additional file 2: Table S3. Unique differentially expressed proteins in VPP compared with VPW. (XLSX 21 kb) Additional file 3: Table S4. Unique differentially expressed proteins in VPE compared with VPW. (DOCX 39 kb) Additional file 4: Table S5. Unique differentially expressed proteins in VPX compared with VPW. (DOCX 65 kb) Additional file 5: Table S6. The correlation of expression between proteins and genes. (XLSX 16 kb)

## Abbreviations

iTRAQ: isobaric tags for relative and absolute quantitation
LC-MS/MS: liquid chromatography-tandem mass spectrometry
qRT-PCR: quantitative real time polymerase chain reaction
TDH: thermostable direct hemolysin
MS: Mass spectrometry
FDR: false discovery rate
GO: Gene Ontology
T3SS: type III secretion system
TRH: TDH-related hemolysin
SD: standard deviation
