# Supplementary material for: iTRAQ-based proteomic profiling of Vibrio parahaemolyticus under various culture conditions
Source: Proteome Sci. 2015 Jul 29;13:19. doi: 10.1186/s12953-015-0075-4 (PMC4518887; doi:10.1186/s12953-015-0075-4)
Supplement: Additional file 2: Table S3. — Unique differentially expressed proteins in VPP compared with VPW. (XLSX 21 kb) [file 12953_2015_75_MOESM2_ESM.xlsx]

**Table S3 Unique differentially expressed proteins in VPP compared with VPW**

| **Accession^a^** | **Protein name** | **Gene** | **MW (kDa)** | **iTRAQ ratio^b^** | **P Value^c^** | **GO** |
| --- | --- | --- | --- | --- | --- | --- |
| **Increased proteins** | | | | | | |
| Q87RJ7 | Cysteine synthase | VP0797 | 34.113 | 2.0324 | 0.0084 | cysteine biosynthetic process from serine |
| L0HRF8 | Peptidase B | pepB | 46.612 | 2.0893 | 0.0353 | proteolysis |
| E1DLQ7 | "2',3'-cyclic-nucleotide 2'-phosphodiesterase | cpdB | 72.335 | 3.0761 | 0.0057 | nucleotide catabolic process |
| F3RPY6 | Membrane-fusion protein |  | 40.162 | 2.7040 | 0.0488 | transmembrane transport |
| L0HZQ5 | "Scaffold protein for [4Fe-4S] cluster assembly ApbC, MRP-like protein | VPBB_1902 | 38.930 | 2.3335 | 0.0282 | ATP binding |
| L0I0A1 | Oxidoreductase short-chain family protein | VPBB_A0116 | 25.865 | 2.0324 | 0.0485 | oxidation-reduction process |
| F3RRY7 | 2-succinyl-5-enolpyruvyl-6-hydroxy-3-cyclohexene-1-carboxylate synthase | menD | 62.798 | 2.1086 | 0.0079 | menaquinone biosynthetic process |
| 87GW3 | Polyhydroxyalkanoicacid synthase | VPA1202 |  | 2.1478 | 0.0398 | poly-hydroxybutyrate  biosynthetic process |
| L0HS29 | Bifunctional protein FolD | folD | 30.806 | 2.2284 | 0.0377 | methenyltetrahydrofolate cyclohydrolase activity,; |
| L0HXG6 | "ABC-type antimicrobial peptide transport system, permease component | VPBB_1834 | 43.926 | 6.2517 | 0.0155 | integral to membrane;  plasma membrane |
| F3RZ16 | Putative uncharacterized protein | VP10329_06702 | 549.134 | 3.8019 | 0.0218 | no GO |
| Q87MZ6 | ABC transporter substrate-binding protein | VP2080 | 33.195 | 2.4889 | 0.0140 | no GO |
| F3RSH1 | uncharacterized protein | VP10329_19140 | 56.918 | 2.5351 | 0.0421 | no GO |
| Q87JY7 | Uncharacterized protein | VPA0111 | 20.760 | 4.7424 | 0.0081 | no GO |
| Q938U8 | Putative uncharacterized protein |  | 106.783 | 5.2000 | 0.0150 | no GO |
| **Decreased proteins** | | | | | | |
| Q87RF0 | 2-oxoglutarate dehydrogenase, E1 component | VP0847 | 106.083 | 0.4406 | 0.0003 | tricarboxylic acid cycle |
| E1EK82 | Glycine dehydrogenase [decarboxylating] | gcvP | 104.123 | 0.2291 | 0.0321 | glycine decarboxylation via glycine cleavage system |
| L0HWI9 | Chromosome partition protein MukB | mukB | 170.014 | 0.2965 | 4.74 E-06 | cell cycle; cell division; chromosome segregation  DNA replication |
| F3RTT1 | Acetyl-CoA carboxylase biotin carboxylase subunit | VP10329_23013 | 49.210 | 0.3162 | 0.0103 | acetyl-CoA carboxylase activity |
| L0HVR0 | Glutamate--tRNA ligase | gltX | 53.453 | 0.3664 | 0.0342 | glutamyl-tRNA aminoacylation |
| Q9ALY5 | Cyclic AMP receptor protein | VP2793 | 23.665 | 0.4446 | 0.0021 | transcription, DNA-dependent |
| Q87G49 | " ATP-dependent RNA helicase, DEAD box family ATP依赖性RNA | VPA1468 | 50.475 | 0.3251 | 0.0058 | ATP-dependent helicase activity |
| Q87SL8 | RNA polymerase sigma factor | VP0404 | 70.711 | 0.4446 | 0.0061 | DNA-dependent transcription, initiation |
| L0I139 | Outer membrane protein A （OMPA） | VPBB_A0227 | 35.568 | 0.4285 | 0.0139 | cell outer membrane |
| Q87TM8 | ATP-dependent DNA helicase Rep | VP0041 | 77.353 | 0.4786 | 0.0498 | DNA unwinding involved in DNA replication |
| L0HYL9 | "Ribonucleotide reductase of class Ia (Aerobic), beta subunit | VPBB_1776 | 43.729 | 0.3162 | 0.0015 | deoxyribonucleoside diphosphate metabolic process |
| Q87TR2 | "Amino acid ABC transporter, ATP-binding protein | VP0006 | 27.293 | 0.1959 | 0.0397 | ATP catabolic process |
| L0HTL2 | Lipoyl synthase | lipA | 36.393 | 0.4446 | 0.0096 | protein lipoylation |
| Q87PX2 | Putative HD-GYP domain containing protein | VP1378 | 44.883 | 0.3499 | 0.0109 | metabolic process |
| Q87KC3 | Thiol:disulfide interchange protein | VP3054 | 22.820 | 0.3565 | 0.0133 | cell redox homeostasis |
| Q87G73 | Putative transport protein | VPA1444 | 63.478 | 0.0752 | 0.0328 | protein secretion by the type I secretion system |
| Q87FS5 | Putative phosphatase | VPA1603 | 16.083 | 0.0946 | 0.0344 | peptidyl-tyrosine dephosphorylation |
| L0HZ61 | Fumarate reductase subunit C | frdC | 14.624 | 0.4169 | 0.0450 | integral to membrane |

a. Protein information including accession numbers, gene names, MW and Gene Ontology (GO) was taken from the UniProtKB

b. iTRAQ ratio of VPP compared with VPW

c. Statistical analysis of iTRAQ ratio of VPP compared with VPW was performed using unpaired t-test.
