# Supplementary material for: iTRAQ-based proteomic profiling of Vibrio parahaemolyticus under various culture conditions
Source: Proteome Sci. 2015 Jul 29;13:19. doi: 10.1186/s12953-015-0075-4 (PMC4518887; doi:10.1186/s12953-015-0075-4)
Supplement: Additional file 3: Table S4. — Unique differentially expressed proteins in VPE compared with VPW. (DOCX 39 kb) [file 12953_2015_75_MOESM3_ESM.docx]

**Table S4 Unique differentially expressed proteins in VPE compared with VPW**

| Accession^a^ | | Protein name | Gene | MW (kDa) | iTRAQ ratio^b^ | P Value^c^ | GO |
| --- | --- | --- | --- | --- | --- | --- | --- |
| **Increased proteins** | | | | | | | |
| L0I1Q9 | | ATP synthase subunit beta | atpD | 50.696 | 2.5823 | 0.0037 | ATP hydrolysis coupled proton transport |
| F3RS00 | | Trigger factor | tig | 48.255 | 3.3729 | 0.0002 | cell cycle, cell division |
| L0HRV5 | | DNA gyrase subunit B | gyrB | 89.442 | 2.3121 | 0.0147 | DNA-dependent DNA replication |
| A6B133 | | Transcription-repair coupling factor | mfd | 130.474 | 2.2080 | 0.0046 | DNA repair |
| L0HTD3 | | GMP synthase [glutamine-hydrolyzing] | guaA | 57.672 | 2.4889 | 0.0014 | GMP biosynthetic process |
| L0HX50 | | GMP synthase [glutamine-hydrolyzing] | VPBB_0973 | 97.996 | 2.5586 | 0.0017 | chromosome |
| L0HSW7 | | 30S ribosomal protein S9 | rpsI | 14.614 | 2.5119 | 0.0015 | translation |
| L0HW03 | | Translation initiation factor IF-2 | infB | 99.396 | 3.73259 | 0.0001 | GTP catabolic process |
| Q87JZ6 | | Putative isomerase | VPA0102 | 100.577 | 3.34199 | 0.0067 | metabolic process |
| A6BBF2 | | Ribosomal protein S7 (Fragment) | rpsG | 17.409 | 2.2491 | 0.0005 | translation |
| F3RSW8 | | Phenylalanine--tRNA ligase beta subunit | pheT | 87.088 | 2.3335 | 0.0040 | phenylalanyl-tRNA aminoacylation |
| Q87M01 | | N utilization substance protein A | VP2457 | 54.945 | 2.0701 | 0.0174 | regulation of DNA-dependent transcription, termination |
| L0HYJ6 | | Ribosomal RNA small subunit methyltransferase I | rlmI | 44.454 | 2.7290 | 2.43E-05 | rRNA (cytosine) methyltransferase activity |
| L0HV54 | | 30S ribosomal protein S10 | rpsJ | 11.721 | 3.5975 | 0.0052 | translation |
| A6B817 | | Cysteinyl-tRNA synthetase (Fragment) | cysS | 52.418 | 2.1878 | 0.0119 | cysteinyl-tRNA aminoacylation |
| L0HSH4 | | 50S ribosomal protein L6 | rplF | 18.779 | 3.3113 | 0.0010 | translation |
| L0HV66 | | 30S ribosomal protein S8 | rpsH | 14.008 | 4.6132 | 0.0086 | translation |
| L0HVZ0 | | Peptide chain release factor 3 | prfC | 59.245 | 2.2699 | 0.0024 | regulation of translational termination |
| Q87P08 | | Glucose-6-phosphate 1-dehydrogenase | VP1710 | 57.640 | 2.6792 | 0.0142 | pentose-phosphate shunt |
| Q87R87 | | C4-dicarboxylate-binding periplasmic protein | VP0910 | 37.132 | 3.7670 | 0.0060 | transport |
| L0HW62 | | Thermostable carboxypeptidase 1 | VPBB_1603 | 55.079 | 4.7863 | 0.0013 | proteolysis |
| L0HUI1 | | 30S ribosomal protein S11 30S | rpsK | 13.890 | 2.4660 | 0.0472 | translation |
| L0HZB1 | | Uroporphyrinogen decarboxylase | hemE | 39.177 | 3.2509 | 0.0288 | protoporphyrinogen IX biosynthetic process |
| E1DBS5 | | Molecular chaperone | VIPARAQ4037_2547 | 70.879 | 4.4055 | 0.0012 | ATP binding |
| L0HQN2 | | 30S ribosomal protein S13 | rpsM | 13.291 | 2.1878 | 0.0424 | translation |
| L0HYY1 | | 50S ribosomal protein L9 | rplI | 15.709 | 2.9648 | 0.0028 | structural constituent of ribosome |
| Q87I01 | | Aminomethyltransferase | VPA0805 | 40.322 | 2.2699 | 0.0291 | glycine catabolic process |
| F3RPN2 | | Electron transport complex protein RnfC | VP10329_17710 | 97.730 | 2.5586 | 0.0336 | electron carrier activity |
| E1EMD9 | Phenylalanine--tRNA ligase alpha subunit | | pheS | 36.873 | 2.6303 | 0.0162 | phenylalanyl-tRNA aminoacylation |
| Q87SS1 | Putative long-chain-fatty-acid-CoA ligase | | VP0351 | 67.920 | 2.8840 | 0.0260 | F:ligase activity |
| L0I0X0 | 30S ribosomal protein S6 | | rpsF | 14.983 | 2.0893 | 0.0050 | translation |
| F3RU57 | Shikimate kinase | | aroK | 19.472 | 2.8576 | 0.0035 | chorismate biosynthetic process |
| L0HVL1 | 50S ribosomal protein L13 | | rplM | 15.961 | 2.1878 | 0.0118 | translation |
| L0HZJ5 | 30S ribosomal protein S18 | | rpsR | 8.843 | 2.9648 | 0.0107 | translation |
| Q87N59 | Uncharacterized protein | | VP2016 | 38.894 | 3.5645 | 0.0004 | iron ion binding |
| F3RPW0 | Orotidine 5'-phosphate decarboxylase | | pyrF | 25.293 | 2.3335 | 0.0034 | ‘de novo’ UMP biosynthetic process |
| Q87JA9 | Putative multidrug resistance protein | | VPA0344 | 117.252 | 2.0893 | 0.0105 | transporter activity |
| L0HWB3 | Ribonuclease 3 | | rnc | 25.068 | 2.4210 | 0.0377 | RNA processing |
| L0HTN3 | Peptide chain release factor 1 | | prfA | 40.294 | 2.7040 | 0.0201 | translation release factor activity, codon specific |
| L0HYK1 | UDP-N-acetylglucosamine 1-carboxyvinyltransferase | | murA | 44.110 | 2.3550 | 0.0180 | cell division |
| F3RRN2 | tRNA (mo5U34)-methyltransferase | | cmoB | 37.104 | 3.8726 | 0.0025 | tRNA wobble uridine modification |
| L0HSP1 | Ubiquinone/menaquinone biosynthesis methyltransferase ubiE | | cmoB | 28.965 | 2.4889 | 0.0235 | ubiquinone biosynthetic process |
| Q87FS4 | Putative tyrosine kinase | | VPA1604 | 78.999 | 4.6989 | 0.0199 | regulation of catalytic activity |
| L0HW25 | Dual-specificity RNA methyltransferase RlmN | | rlmN | 42.170 | 2.7290 | 0.0292 | rRNA base methylation |
| Q87QH8 | "Peptide ABC transporter, periplasmic peptide-binding protein | | VP1171 | 60.934 | 2.3121 | 0.0375 | transporter activity |
| L0HX06 | Oligopeptide transport ATP-binding protein OppD | | VPBB_1924 | 36.035 | 3.7670 | 0.0248 | ATP catabolic process |
| L0HUF9 | 50S ribosomal protein L31 | | rpmE | 8.055 | 3.6983 | 0.0276 | translation |
| L0HYV4 | 50S ribosomal protein L19 | | rplS | 13.219 | 3.4041 | 0.0333 | structural constituent of ribosome |
| Q87HH8 | "Nitrite reductase (NAD(P)H), large subunit | | VPA0987 | 93.572 | 4.5290 | 0.0269 | nitrate assimilation |
| Q87PU8 | Uncharacterized protein | | VP1403 | 55.577 | 9.6383 | 1.7E-11 | no GO |
| Q87T55 | Putative OtnG protein | | VP0215 | 83.085 | 2.1281 | 0.0071 | no GO |
| L0HWA8 | Decarboxylase family protein | | VPBB_0669 | 50.596 | 2.0701 | 0.0403 | no GO |
| Q87PU9 | Uncharacterized protein | | VP1402 | 18.567 | 14.7231 | 8.23E-05 | no GO |
| L0I1Z3 | UPF0319 protein VPBB_A1448 | | VPBB_A1448 | 24.348 | 2.2699 | 0.0413 | no GO |
| Q87LF1 | Uncharacterized protein | | VP2661 | 24.076 | 2.2699 | 0.0280 | no GO |
| Q87PV3 | Uncharacterized protein | | VP1398 | 37.532 | 2.7040 | 0.0032 | no GO |
| F3RSG6 | Putative uncharacterized protein (Fragment) | | VP10329_19115 | 284.350 | 2.8314 | 0.0013 | no GO |
| E1EMQ2 | "Type VI secretion protein, VC_A0110 family | | VIPARK5030_1375 | 65.705 | 2.8840 | 0.0173 | no GO |
| Q87PW1 | Uncharacterized protein | | VP1389 | 43.372 | 2.2909 | 0.0462 | no GO |
| Q87JW0 | Putative TldD protein | | VPA0138 | 49.479 | 2.0512 | 0.0280 | no GO |
| Q87PW2 | Uncharacterized protein | | VP1388 | 65.964 | 3.9811 | 0.0160 | no GO |
| Q87PU8 | Putative OtnG protein | | VP1403 | 55.577 | 9.6383 | 1.7E-11 | no GO |
| Q87T55 | Decarboxylase family protein | | VP0215 | 83.085 | 2.1281 | 0.0071 | no GO |
| L0HWA8 | Uncharacterized protein | | VPBB_0669 | 50.596 | 2.0701 | 0.0403 | no GO |
| **Decreased proteins** | | | | | | | |
| E1D6T7 | Oxaloacetate decarboxylase alpha subunit | | oadA | 64.204 | 0.1977 | 0.0012 | metabolic process |
| Q87RF5 | Citrate synthase | | VP0842 | 48.219 | 0.4656 | 0.0204 | tricarboxylic acid cycle |
| L0I2S8 | Heme transport protein HutA | | VPBB_A0827 | 77.240 | 0.0437 | 2.13 E-07 | transporter activity  receptor activity |
| E1EF47 | Fatty acid oxidation complex subunit alpha | | fadB | 78.556 | 0.1690 | 2.15E-05 | fatty acid beta-oxidation |
| Q87MM5 | "Peptidase, insulinase family | | VP2206 | 105.746 | 0.4365 | 0.0049 | proteolysis |
| F3RTF5 | Putative uncharacterized protein | | VP10329_12136 | 64.577 | 0.2992 | 0.0002 | hydrolase activity, acting on carbon-nitrogen (but not peptide) bonds |
| E1DBF1 | "Oligoendopeptidase, PepF/M3 family | | VIPARAQ4037_2676 | 67.958 | 0.3873 | 0.0036 | proteolysis |
| Q87FE3 | Toxin secretion ATP-binding protein | | VPA1736 | 78.109 | 0.3532 | 2.6E-05 | proteolysis |
| L0HXD2 | Fatty acid oxidation complex subunit alpha | | fadJ | 76.796 | 0.3436 | 0.0001 | fatty acid beta-oxidation |
| L0HY24 | 3-ketoacyl-CoA thiolase | | fadI | 46.396 | 0.3251 | 0.0008 | fatty acid beta-oxidation |
| Q87LK9 | Iron-regulated outer membrane virulence protein homolog | | VP2602 | 71.948 | 0.1459 | 0.0002 | transporter activity,receptor activity |
| F3RTF4 | Homocysteine synthase | | VP10329_12131 | 45.746 | 0.2559 | 0.0010 | transferase activity, transferring alkyl or aryl (other than methyl) groups |
| Q87P87 | Agglutination protein | | VP1631 | 50.705 | 0.1343 | 0.0002 | protein transport |
| A6AYD5 | Catalase-peroxidase | | katG | 82.177 | 0.4656 | 0.0339 | hydrogen peroxide catabolic process |
| Q87JU6 | Biopolymer transport protein ExbB-related protein | | VPA0152 | 49.200 | 0.0637 | 0.0041 | protein transporter activity |
| F3S0P9 | D-alanyl-D-alanine carboxypeptidase | | VP10329_10876 | 42.204 | 0.3076 | 0.0032 | proteolysis |
| Q87M39 | Putative pilus assembly transmembrane protein | | VP2419 | 52.723 | 0.1096 | 0.0139 | protein secretion |
| Q87SA3 | Phosphoenolpyruvate-protein phosphotransferase | | VP0521 | 83.774 | 0.3565 | 0.0201 | phosphoenolpyruvate-dependent sugar phosphotransferase system |
| Q87L24 | Phosphoribulokinase | | VP2792 | 32.758 | 0.3945 | 0.0282 | carbohydrate metabolic process |
| L0HU11 | D-amino acid dehydrogenase small subunit | | dadA | 46.433 | 0.4613 | 0.0420 | D-alanine catabolic process |
| E1EF46 | 3-ketoacyl-CoA thiolase | | fadA | 41.406 | 0.3631 | 0.0093 | fatty acid beta-oxidation |
| Q87PG7 | Putative stress protein | | VP1535 | 35.330 | 0.2582 | 0.0200 | ciliary or bacterial-type flagellar motility |
| Q87K27 | Putative alcohol dehydrogenase | | VPA0071 | 40.776 | 0.4831 | 0.0011 | ethanol oxidation |
| L0I2K8 | Cyclohexadienyl dehydratase | | VPBB_2914 | 28.958 | 0.3532 | 0.0030 | transporter activity |
| L0HW43 | Uncharacterized protein | | VPBB_2322 | 86.211 | 0.4875 | 0.0475 | lipid metabolic process |
| Q87JV2 | Putative glycerophosphoryl diester phosphodiesterase | | VPA0146 | 26.666 | 0.4656 | 0.0284 | lipid metabolic process |
| E1D737 | "Exodeoxyribonuclease V, gamma subunit | | recC | 132.333 | 0.4365 | 0.0163 | exodeoxyribonuclease V activity |
| Q87H59 | Putative stomatin-like protein | | VPA1106 | 29.596 | 0.2421 | 0.0038 | membrane |
| A6B3F3 | "ABC transporter, ATP-binding protein | | A79_1175 | 28.661 | 0.3499 | 0.0339 | ATP catabolic process |
| A6B1P9 | 短链脱氢酶 | | mmsA | 54.501 | 0.1754 | 0.0050 | methylmalonate-semialdehyde dehydrogenase (acylating) activity |
| L0HV38 | Uncharacterized protein | | VPBB_1962 | 44.080 | 0.2333 | 0.0100 | phosphorelay signal transduction system |
| Q87I45 | Aldehyde dehydrogenase | | VPA0761 | 52.387 | 0.4742 | 0.0260 | cellular aldehyde metabolic process |
| Q87HT7 | GGDEF family protein | | VPA0869 | 72.334 | 0.2884 | 0.0060 | intracellular signal transduction |
| F3RNI4 | Cell division protein ZapD | | zapD | 28.621 | 0.4529 | 0.0476 | barrier septum assembly |
| L0HZ08 | Pyridoxamine 5'-phosphate oxidase-related putative heme iron utilization protein | | VPBB_A0398 | 20.191 | 0.1127 | 0.0403 | pyridoxal phosphate biosynthetic process |
| Q87IE2 | Putaive Fe-regulated protein B | | VPA0664 | 74.206 | 0.1871 | 0.0378 | siderophore transmembrane transport |
| L0I331 | TonB system biopolymer transport component Chromosome segregation ATPase | | VPBB_A0140 | 28.151 | 0.0817 | 0.0003 | no GO |
| A6B121 | UPF0229 protein A79_6085 | | A79_6085 | 49.937 | 0.1585 | 5.24E-08 | no GO |
| Q87H91 | Uncharacterized protein | | VPA1074 | 35.652 | 0.2992 | 0.0011 | no GO |
| L0I1J4 | Periplasmic hemin-binding protein | | VPBB_A0392 | 30.236 | 0.0847 | 0.04411 | no GO |
| L0I2C0 | Putative nonspecific acid phosphatase | | VPBB_A0642 | 37.157 | 0.2443 | 0.0066 | no GO |
| F3RPL8 | Short chain dehydrogenase | | VP10329_17640 | 27.237 | 0.1500 | 0.0110 | no GO |
| L0I0Q7 | Putative exported protein | | VPBB_A0097 | 49.135 | 0.2679 | 0.0111 | no GO |
| Q87NJ7 | Uncharacterized protein | | VP1871 | 51.784 | 0.4571 | 0.0331 | no GO |
| F3RSV7 | Putative uncharacterized protein | | VP10329_19830 | 25.845 | 0.3311 | 0.0183 | no GO |
| Q87KC8 | SpoOM-related protein | | VP3049 | 30.311 | 0.1837 | 0.0077 | no GO |
| Q87G54 | Uncharacterized protein | | VPA1463 | 20.786 | 0.0299 | 0.0264 | no GO |
| L0HZB2 | Iron-regulated protein A | | VPBB_1761 | 45.388 | 0.2208 | 0.0212 | no GO |

a. Protein information including accession numbers, gene names, MW and Gene Ontology (GO) was taken from the UniProtKB

b. iTRAQ ratio of VPP compared with VPW

c. Statistical analysis of iTRAQ ratio of VPP compared with VPW was performed using unpaired t-test.
