# Supplementary material for: iTRAQ-based proteomic profiling of Vibrio parahaemolyticus under various culture conditions
Source: Proteome Sci. 2015 Jul 29;13:19. doi: 10.1186/s12953-015-0075-4 (PMC4518887; doi:10.1186/s12953-015-0075-4)
Supplement: Additional file 4: Table S5. — Unique differentially expressed proteins in VPX compared with VPW. (DOCX 65 kb) [file 12953_2015_75_MOESM4_ESM.docx]

**Table S5 Unique differentially expressed proteins in VPX compared with VPW**

| **Accession^a^** | **Protein name** | **Gene** | **MW (kDa)** | **iTRAQ ratio^b^** | **P Value^c^** | **GO** |
| --- | --- | --- | --- | --- | --- | --- |
| **Increased proteins** | | | | | | |
| Q87LU2 | Pyruvate dehydrogenase component | VP2519 | 99.664 | 2.6792 | 0.0002 | pyruvate dehydrogenase (acetyl-transferring) activity |
| F3RV73 | Carbamoyl-phosphate synthase large chain | carB | 119.473 | 4.8753 | 2.88E-10 | ‘de novo' UMP biosynthetic process,  arginine biosynthetic process |
| L0HVR5 | Glutamate synthase [NADPH] large chain | VPBB_0461 | 163.087 | 12.0226 | 1.18E-09 | glutamate biosynthetic process |
| Q87MW0 | Alcohol dehydrogenase/acetaldehyde dehydrogenase | VP2121 | 97.123 | 4.2462 | 0.0014 | alcohol metabolic process |
| L0HYW5 | "Protease, insulinase family | VPBB_2362 | 105.826 | 2.6062 | 1.82E-08 | proteolysis |
| G1C7F6 | Alanine--tRNA ligase |  |  | 10.0925 | 0.0072 | carbohydrate transport  ion transport |
| E1EM82 | "Succinate dehydrogenase, flavoprotein subunit | purL | 141.687 | 6.6069 | 5.17E-09 | ‘de novo' IMP biosynthetic process,glutamine metabolic process |
| F3RRL6 | Protein TolB | tolB | 49.824 | 3.6983 | 4.85E-08 | protein import |
| F3RU41 | Phosphoenolpyruvate carboxylase | ppc | 99.277 | 3.3729 | 6.44 E-07 | tricarboxylic acid cycle |
| L0HZF1 | Sulfite reductase [NADPH] hemoprotein beta-component | cysI | 64.848 | 5.3456 | 4.22E-09 | cysteine biosynthetic process  sulfate assimilation |
| L0HWS0 | "6-phosphogluconate dehydrogenase, decarboxylating | VPBB_1568 | 52.563 | 4.1687 | 0.0006 | D-gluconate metabolic process |
| Q87HP1 | NAD(P) transhydrogenase subunit alpha | VPA0922 | 54.806 | 3.8371 | 1.06 E-06 | proton transport |
| F3RSR6 | Oligopeptide ABC transporter periplasmic oligopeptide-binding protein | VP10329_19625 | 60.239 | 3.8019 | 0.0001 | transporter activity |
| Q87P15 | Aldehyde dehydrogenase | VP1703 | 55.686 | 6.0813 | 0.0001 | oxidoreductase activity, acting on the aldehyde or oxo group of donors, NAD or NADP as acceptor |
| Q87KJ3 | Diaminopimelate decarboxylase | lysA | 45.890 | 3.3113 | 0.0010 | lysine biosynthetic process via diaminopimelate |
| Q87SS0 | Acetolactate synthase | VP0352 | 63.046 | 7.5162 | 2.61 E-06 | isoleucine biosynthetic process,  valine biosynthetic process |
| Q87FQ8 | Phosphorylase | VPA1620 | 92.400 | 5.2481 | 1.19 E-07 | carbohydrate metabolic process |
| F3RUD3 | Putative sigma-54 interacting response regulator transcription regulator protein | VP10329_00700 | 50.312 | 2.6546 | 0.0002 | regulation of transcription, DNA-dependent |
| L0HZ29 | UvrABC system protein B | uvrB | 77.043 | 2.2699 | 0.0144 | nucleotide-excision repair |
| Q87PH6 | Spermidine/putrescineABCtransporter,periplasmic spermidine/putrescine-binding protein | VP1526 | 39.683 | 4.0926 | 0.0001 | polyamine transport |
| Q87Q15 | Probable dihydrodipicolinate synthetase | VP1335 | 33.957 | 10.1859 | 0.0228 | lyase activity |
| L0HZL7 | Aspartokinase [ə,spɑːtəʊ'kaɪneɪz] | VPBB_2621 | 87.761 | 2.1281 | 0.0073 | aspartate family amino acid biosynthetic process |
| L0I440 | Maltose operon periplasmic protein MalM | VPBB_A1506 | 29.939 | 5.6494 | 1.5 E-05 | carbohydrate transport |
| Q87MP6 | Amidophosphoribosyltransferase | VP2185 | 56.045 | 5.9704 | 0.0007 | nucleoside metabolic process |
| F3RP38 | [Protein-PII] uridylyltransferase | glnD | 100.221 | 2.2080 | 0.0346 | nitrogen compound metabolic process |
| F3RXY2 | 3-ketoacyl-(Acyl-carrier-protein) reductase | fabG | 26.824 | 5.0119 | 0.0003 | 3-oxoacyl-[acyl-carrier-protein] reductase (NADPH) activity |
| E1DJZ3 | Exoribonuclease 2 | rnb | 75.438 | 2.1086 | 0.0032 | nucleic acid phosphodiester bond hydrolysis |
| L0HTB3 | Protein translocase subunit SecD | secD | 65.387 | 2.3988 | 0.0097 | protein transport by the Sec complex |
| E1EFN4 | Tryptophan biosynthesis protein trpCF | VIPARK5030_1904 | 52.714 | 5.7016 | 2.48 E-05 | tryptophan biosynthetic process |
| F3RPY6 | Membrane-fusion protein | VP10329_18230 | 40.162 | 8.8716 | 0.0034 | transmembrane transport |
| A6AZ80 | Acyl-CoA dehydrogenase | A79_4492 | 41.978 | 8.0910 | 4.39 E-05 | acyl-CoA dehydrogenase activity,  flavin adenine dinucleotide binding |
| L0HWJ4 | Aspartate carbamoyltransferase regulatory chain | pyrI | 17.405 | 2.8054 | 0.0342 | pyrimidine nucleotide biosynthetic process |
| F3RXL7 | Tyrosine--tRNA ligase | tyrS | 46.697 | 3.6308 | 0.0009 | pyrimidine nucleotide biosynthetic process |
| L0HVZ7 | Recombination-associated protein RdgC | rdgC | 34.297 | 2.7542 | 0.0233 | DNA recombination |
| L0HV74 | T-protein | VPBB_0521 | 42.032 | 3.1915 | 0.0062 | tyrosine biosynthetic process |
| E1DEC9 | Acetoacetate-CoA ligase | VIPARAQ4037_A0535 | 73.881 | 18.1970 | 1.56 E-05 | lipid metabolic process |
| Q87QR1 | Putative chemotaxis transducer | VP1088 | 59.073 | 2.5586 | 0.0009 | signal transducer activity |
| L0I4N3 | Methylcrotonyl-CoA carboxylase carboxyl transferase subunit | VPBB_A1029 | 58.239 | 3.5645 | 0.0004 | transferase activity |
| E1EFW8 | Methyl-accepting chemotaxis protein | VIPARK5030_0929 | 81.174 | 2.6303 | 0.0053 | signal transducer activity |
| Q87H17 | Putative high-affinity branched-chain amino acid transport ATP-binding protein | VPA1148 | 30.614 | 6.0813 | 2.34 E-05 | ATP catabolic process |
| L0I632 | Transport ATP-binding protein MalK | VPBB_A1275 |  | 5.6494 | 0.0060 | ATP catabolic process |
| Q87HP2 | NAD(P) transhydrogenase subunit beta | VPA0921 | 41.157 | 2.8840 | 0.0036 | NADP binding |
| L0I6K2 | Periplasmic alpha-amylase | VPBB_A1480 | 76.956 | 2.8840 | 0.0020 | alpha-glucan catabolic process |
| P22099 | Anthranilate synthase component 1 | trpE | 59.771 | 7.3790 | 0.0045 | tryptophan biosynthetic process |
| Q87KR4 | Uncharacterized protein | VP2912 | 41.683 | 3.9446 | 0.0148 | adenyl nucleotide binding |
| B8K898 | Anthranilate phosphoribosyltransferase | trpD | 35.440 | 3.9811 | 0.0002 | tryptophan biosynthetic process |
| Q87MS9 | Protease IV | VP2152 | 67.510 | 2.8054 | 0.0221 | proteolysis |
| L0I0H5 | Putative deacylase | VPBB_A0923 | 36.175 | 5.8614 | 0.0007 | metal ion binding |
| L0I414 | UPF0312 protein VPBB_A0794 | VPBB_A0794 | 20.296 | 4.1305 | 0.0015 | periplasmic space |
| L0I3F0 | Electron transfer flavoprotein alpha subunit | VPBB_A1057 | 32.456 | 5.2000 | 0.0037 | flavin adenine dinucleotide binding |
| L0I3C9 | Methyl-accepting chemotaxis protein | VPBB_A0559 | 49.030 | 4.8306 | 0.0006 | phosphorelay sensor kinase activity  P:signal transduction by phosphorylation |
| Q9LB17 | Flagellar biosynthesis protein FlhF | flhF | 56.297 | 2.3768 | 0.0056 | P:bacterial-type flagellum organization |
| Q87TE3 | Uncharacterized protein | VP0127 | 34.792 | 6.3096 | 0.0010 | N-acetyltransferase activity |
| F3RPJ1 | Alkaline phosphatase | VP10329_17495 | 57.407 | 24.2103 | 0.0014 | dephosphorylation |
| Q87S54 | Uncharacterized protein | VP0570 | 49.721 | 5.1523 | 0.0001 | regulation of transcription, DNA-dependent |
| L0I0Y5 | Argininosuccinate lyase | argH | 69.358 | 15.4170 | 0.0020 | arginine biosynthetic process via ornithine |
| L0HSJ2 | Sulfate adenylyltransferase subunit 2 | cysD | 34.983 | 2.2699 | 0.0139 | sulfate assimilation,sulfate reduction |
| L0I0Q1 | Phosphoserine phosphatase | VPBB_2257 | 35.816 | 2.1677 | 0.0341 | dephosphorylation |
| A6AZ75 | Isovaleryl-CoA dehydrogenase | A79_4487 | 42.405 | 11.0662 | 0.0183 | acyl-CoA dehydrogenase activity,  flavin adenine dinucleotide binding |
| Q87IU5 | Methyl-accepting chemotaxis protein | VPA0511 | 67.873 | 9.9083 | 0.0006 | signal transducer activity |
| Q87G08 | ScrC (Sensory box/GGDEF family protein) (Involved in swarmer cell regulation) | VPA1511 | 88.233 | 3.1046 | 0.0071 | cyclic nucleotide biosynthetic process,  intracellular signal transduction |
| Q87M78 | "2,3,4,5-tetrahydropyridine-2,6-dicarboxylate N-succinyltransferase | dapD | 35.640 | 3.5645 | 0.0028 | diaminopimelate biosynthetic process,  lysine biosynthetic process via diaminopimelate |
| F3RXL6 | "Spermidine/putrescine ABC transporter, periplasmic spermidine/putrescine-binding protein | VP10329_05327 | 38.693 | 5.0119 | 0.0031 | polyamine binding |
| L0HUQ9 | Uncharacterized protein | VPBB_0098 | 16.476 | 2.0701 | 0.0481 | N-acetyltransferase activity |
| L0HVD8 | 2-isopropylmalate synthase | leuA | 56.141 | 2.3988 | 0.0224 | leucine biosynthetic process |
| Q87H10 | Electron transfer flavoprotein-ubiquinone oxidoreductase | VPA1155 | 60.326 | 6.3096 | 0.0130 | electron-transferring-flavoprotein dehydrogenase activity,  iron-sulfur cluster binding |
| Q9LB16 | Site-determining protein | flhG | 32.075 | 2.2909 | 0.0044 | ATP binding |
| Q87ME4 | Putative membrane-associated Zn-dependent protease | VP2311 | 49.153 | 6.0256 | 0.0047 | proteolysis |
| F3RNY8 | Murein transglycosylase A | mltA | 40.453 | 3.9084 | 0.0056 | peptidoglycan turnover |
| Q87L50 | Cystathionine gamma-synthase | VP2765 | 41.862 | 4.5290 | 0.0057 | metabolic process |
| L0I2F2 | tRNA(Met) cytidine acetyltransferase TmcA | tmcA | 75.808 | 2.5823 | 0.0117 | tRNA wobble cytosine modification |
| L0I0L8 | Magnesium and cobalt transport protein CorA | VPBB_2217 | 36.294 | 8.2414 | 0.0076 | metal ion transmembrane transporter activity |
| Q87SR9 | "Acetolactate synthase III, small subunit | VP0353 | 18.252 | 4.3652 | 0.0072 | branched-chain amino acid biosynthetic process |
| L0HUR7 | "Type I secretion outer membrane protein, TolC | VPBB_1835 | 46.578 | 9.2045 | 0.0086 | transporter activity |
| L0HU88 | Methionine ABC transporter ATP-binding protein | VPBB_0677 | 37.567 | 8.0168 | 0.0082 | ATP catabolic process |
| L0I2S7 | 3-ketoacyl-CoA thiolase | VPBB_A1026 | 41.765 | 3.1623 | 0.0102 | transferase activity, transferring acyl groups other than amino-acyl groups |
| L0HVC4 | Protease HtpX | htpX | 31.082 | 2.8840 | 0.0268 | Proteolysis,  response to stress |
| Q87KM0 | Uncharacterized protein | VP2957 | 22.451 | 3.1915 | 0.0363 | rRNA methylation |
| A6B0K6 | Acetoacetyl-CoA reductase | A79_3719 | 27.195 | 2.3550 | 0.0337 | poly-hydroxybutyrate biosynthetic process |
| L0HVN4 | Apolipoprotein N-acyltransferase | lnt | 55.281 | 6.9183 | 0.0168 | lipoprotein biosynthetic process |
| L0I2V9 | Electron transfer flavoprotein beta subunit | VPBB_A1056 | 27.455 | 2.6303 | 0.0417 | electron carrier activity |
| Q87M31 | "Putative transcriptional regulator, LysR family | VP2427 | 35.419 | 8.8716 | 0.0041 | transcription, DNA-dependent |
| L0HWI3 | "Transcriptional regulator, MarR family | VPBB_1747 | 22.244 | 3.1046 | 0.0276 | transcription, DNA-dependent |
| L0HYZ7 | Acetylglutamate kinase | argB | 27.107 | 28.5759 | 0.0182 | arginine biosynthetic process |
| E1D807 | Ada regulatory protein | VIPARAQ4037_A0005 | 51.839 | 2.7040 | 0.0149 | transcription, DNA-dependent |
| Q87IL3 | Putative transcriptional regulator | VPA0593 | 27.182 | 4.3652 | 0.0102 | regulation of transcription, DNA-dependent |
| Q9AF12 | ScrB | scrB | 36.052 | 7.0469 | 0.0232 | transporter activity |
| L0HVV7 | "Microbial collagenase, secreted | VPBB_1263 | 89.714 | 6.7920 | 0.0025 | proteolysis |
| Q87JP7 | Malate synthase-related protein | VPA0201 | 21.985 | 24.4343 | 0.0431 | metabolic process |
| Q87P69 | "Transcriptional regulator, GntR family | VP1649 | 27.283 | 5.0582 | 0.0152 | regulation of fatty acid metabolic process,  transcription, DNA-dependent |
| Q87GX4 | Putative multidrug efflux membrane fusion protein | VPA1191 | 40.745 | 5.2966 | 0.0243 | transmembrane transport |
| F3RYQ7 | "ABC transporter, periplasmic substrate-binding protein | VP10329_06422 | 29.776 | 16.4437 | 0.0486 | transport |
| A6AX34 | "Phosphate ABC transporter, permease protein PstA | pstA | 32.052 | 4.6559 | 0.0155 | phosphate ion transmembrane transport |
| L0HYJ7 | Ornithine carbamoyltransferase | VPBB_2473 | 37.316 | 6.5464 | 0.0470 | ornithine carbamoyltransferase activity |
| Q87GL9 | D-alanyl-D-alanine carboxypeptidase | VPA1296 | 42.611 | 8.630 | 0.0196 | proteolysis |
| F3RNX1 | Glycerol uptake facilitator protein GlpF | VP10329_16385 | 30.020 | 2.6792 | 0.0404 | transporter activity |
| L0I4W7 | Putative permease | VPBB_A0820 | 89.845 | 11.0662 | 0.0302 | plasma membrane |
| Q87PV1 | Uncharacterized protein | VP1400 | 77.245 | 3.5645 | 0.0135 | protein serine/threonine kinase activity |
| Q5U9E7 | CHO cell elongating factor | cef | 84.348 | 6.5464 | 0.0003 | no GO |
| Q4ZIM7 | Outer membrane protein | ompU | 36.212 | 7.4473 | 0.0135 | no GO |
| L0HV79 | Uncharacterized protein | VPBB_0990 | 54.869 | 3.6644 | 0.0002 | no GO |
| Q87RS3 | Lipoprotein | VP0704 | 20.907 | 2.5823 | 0.0336 | no GO |
| F3RWJ5 | Putative uncharacterized protein | VP10329_04452 | 73.640 | 14.7231 | 0.0209 | no GO |
| F3S0B6 | Putative uncharacterized protein | VP10329_10211 | 60.906 | 3.3113 | 0.0002 | no GO |
| Q87HU6 | Long-chain fatty acid transport protein | VPA0860 | 44.961 | 6.7920 | 0.0001 | no GO |
| Q87SE3 | "Glutamate synthase, small subunit | VP0481 | 52.515 | 3.8726 | 0.0047 | no GO |
| F3S1M9 | Effector protein | VP10329_14930 | 41.725 | 7.0469 | 0.0350 | no GO |
| Q87MB7 | Uncharacterized protein | VP2339 | 27.553 | 2.0512 | 0.0294 | no GO |
| Q87H69 | Uncharacterized protein | VPA1096 | 18.959 | 4.4463 | 0.0006 | no GO |
| Q87JV5 | Uncharacterized protein | VPA0143 | 15.396 | 2.7542 | 0.0412 | no GO |
| **Decreased proteins** | | | | | | |
| Q87R04 | Formate acetyltransferas | VP0994 | 84.520 | 0.1076 | 1.78E-09 | carbohydrate metabolic process |
| L0HWA4 | Enolase | eno | 45.562 | 0.1343 | 0.0013 | magnesium ion binding,  phosphopyruvate hydratase activity |
| L0HRK0 | Chaperone protein DnaK | dnaK | 69.055 | 0.1009 | 3.22 E-07 | protein folding,  response to stress |
| Q87RF2 | Succinate dehydrogenase, flavoprotein subunit | VP0845 | 64.248 | 0.1722 | 0.0106 | \| electron transport chain, \| \| --- \| \| tricarboxylic acid cycle \| |
| L0HUT2 | Putative formate dehydrogenase large subunit | VPBB_1850 | 113.305 | 0.0263 | 0.0143 | \| oxidoreductase activity \| \| --- \| |
| L0HT54 | Lysine--tRNA ligase | lysS | 57.557 | 0.2399 | 0.0027 | lysyl-tRNA aminoacylation |
| A6B7B1 | N-succinylglutamate 5-semialdehyde dehydrogenase | astD | 52.076 | 0.1528 | 0.0066 | arginine catabolic process to succinate |
| E1EF38 | Glycine--tRNA ligase beta subunit | glyS | 76.199 | 0.2249 | 0.0004 | glycyl-tRNA aminoacylation |
| L0HX52 | Adenylosuccinate synthetase | purA | 47.694 | 0.1148 | 0.0002 | ‘de novo' AMP biosynthetic process |
| L0I272 | Biotin sulfoxide reductase | VPBB_A0119 | 89.861 | 0.0780 | 3.95E-10 | \| electron carrier activity, \| \| --- \| \| oxidoreductase activity \| |
| L0I5J8 | L-lactate dehydrogenase [cytochrome] | lldD | 41.400 | 0.0912 | 0.0025 | lactate metabolic process |
| A6AYY5 | "D-amino acid dehydrogenase, small subunit | A79_6216 | 45,871 | 0.1138 | 0.0001 | oxidoreductase activity |
| Q87IX1 | "Putative formate dehydrogenase, alpha subunit | VPA0485 | 156.954 | 0.1096 | 0.0007 | formate metabolic process |
| L0I2W0 | Ribonucleotide reductase of class III (Anaerobic) large subunit | VPBB_A0862 | 79.612 | 0.0780 | 9.23E-10 | DNA replication |
| L0HSP0 | Serine--tRNA ligase | serS | 48.811 | 0.2051 | 0.0002 | seryl-tRNA aminoacylation |
| L0HU47 | ATP-dependent Clp protease ATP-binding subunit ClpX | clpX | 46.693 | 0.4613 | 0.0146 | proteolysis |
| Q87S44 | "Antioxidant, AhpC/Tsa family | VP0580 | 22.237 | 0.2965 | 0.0003 | antioxidant activity |
| L0I007 | Glycerol-3-phosphate acyltransferase | plsB | 91.143 | 0.3802 | 0.0004 | CDP-diacylglycerol biosynthetic process |
| Q87KW2 | Aspartate ammonia-lyase | VP2863 | 52.733 | 0.1941 | 0.0002 | aspartate metabolic process |
| F3RXT7 | Putative uncharacterized protein | VP10329_05552 | 41.991 | 0.3908 | 0.0006 | transferase activity, transferring acyl groups |
| Q87P84 | Agglutination protein | VP1634 | 48.690 | 0.1107 | 0.0001 | protein transport |
| Q87G09 | 2-amino-3-ketobutyrate coenzyme A ligase | VPA1510 | 43.170 | 0.0550 | 4.75 E-05 | biosynthetic process |
| Q87TR0 | "Amino acid ABC transporter | VP0008 | 27.502 | 0.4742 | 0.0112 | transporter activity |
| Q87LA8 | MSHA biogenesis protein MshL | VP2704 | 58.649 | 0.4446 | 0.0009 | pilus assembly,  protein secretion |
| Q87FP3 | "Ornithine decarboxylase, inducible | VPA1635 | 82.341 | 0.0520 | 0.0005 | cellular amino acid metabolic process |
| Q87RK0 | Phosphoenolpyruvate-protein phosphotransferase | VP0794 | 63.192 | 0.3373 | 0.0183 | phosphoenolpyruvate-dependent sugar phosphotransferase system |
| Q87K22 | Putative regulator | VPA0076 | 30.163 | 0.4875 | 0.0125 | regulation of transcription, DNA-dependent |
| L0HVZ4 | GGDEF & EAL family protein | VPBB_2272 | 77.160 | 0.4018 | 0.0013 | cyclic nucleotide biosynthetic process,  intracellular signal transduction |
| E1DJB0 | "Alcohol dehydrogenase, iron-dependent | VIPARAN5034_0848 | 48.692 | 0.0353 | 1.63 E-05 | oxidoreductase activity,metal ion binding |
| Q87KY4 | "Fumarate reductase, flavoprotein subunit | VP2840 | 66.107 | 0.1514 | 1.15 E-07 | P:anaerobic respiration  electron transport chain |
| E1ECC9 | Urocanate hydratase | hutU | 61.867 | 0.2630 | 0.0066 | P:histidine catabolic process to glutamate and formamide |
| F3RNS4 | Urocanate hydratase | deoB | 44.097 | 0.0920 | 0.0023 | 5-phosphoribose 1-diphosphate biosynthetic process |
| Q87PH7 | "Spermidine/putrescine ABC transporter | VP1525 | 38.999 | 0.2188 | 0.0012 | polyamine transport |
| Q87QQ8 | Putative transmembrane protein affecting septum formation and cell membrane permeability | VP1091 | 40.390 | 0.2606 | 1.76 E-05 | transmembrane transport |
| L0HV37 | Nucleoside-diphosphate sugar epimerase dehydratase | VPBB_0247 | 69.069 | 0.4875 | 0.0122 | biosynthetic process |
| Q87HX7 | Iron-containing alcohol dehydrogenase | VPA0829 | 41.865 | 0.0254 | 0.0002 | oxidoreductase activity |
| A6B9B3 | ABC-type multidrug efflux pump | A79_0807 | 68.489 | 0.2089 | 0.0190 | ATP catabolic process |
| E1DGU5 | 4-hydroxy-3-methylbut-2-en-1-yl diphosphate synthase | ispG | 40.689 | 0.2754 | 0.0079 | isopentenyl diphosphate biosynthetic process |
| L0HVJ1 | "6,7-dimethyl-8-ribityllumazine synthase | ribH | 16.432 | 0.3733 | 0.0249 | riboflavin biosynthetic process |
| Q87L48 | Putative malate oxidoreductase | VP2767 | 46.164 | 0.3048 | 0.0042 | malate metabolic process |
| Q87QB4 | Uncharacterized protein | VP1236 | 31.562 | 0.2270 | 0.0047 | carbohydrate metabolic process |
| L0I1I3 | "Guanosine-5'-triphosphate,3'-diphosphate pyrophosphatase | VPBB_2836 | 52.553 | 0.3311 | 0.0209 | DNA-dependent transcription, initiation |
| L0HY87 | Butyryl-CoA dehydrogenase | VPBB_2105 | 88.618 | 0.1057 | 0.0044 | fatty acid beta-oxidation using acyl-CoA dehydrogenase |
| L0HVP4 | Type cbb3 cytochrome oxidase biogenesis protein CcoI | VPBB_1444 | 85.898 | 0.4365 | 0.0120 | metal ion transport |
| F3RQ86 | Methyl-accepting chemotaxis protein | VP10329_18730 | 59.475 | 0.1905 | 0.0071 | signal transducer activity |
| Q87LC5 | Cytoplasmic axial filament protein | VP2687 | 55.357 | 0.1888 | 0.0446 | RNA processing |
| Q87LM5 | VisC protein | VP2586 | 44.099 | 0.4365 | 0.0376 | ubiquinone biosynthetic process |
| Q87FF4 | Uncharacterized protein | VPA1725 | 25.475 | 0.1941 | 0.0024 | oxidoreductase activity |
| B8K561 | Purine nucleoside phosphorylase DeoD-type | deoD_1 | 61.867 | 0.1380 | 0.0032 | purine nucleoside metabolic process |
| L0HQS3 | 50S ribosomal protein L21 | rplU | 25.642 | 0.4167 | 0.0040 | translation |
| Q87FE7 | Putative two-component response regulator | VPA1732 | 46.667 | 0.3251 | 0.0170 | regulation of transcription, DNA-dependent |
| L0I5K7 | Phospho-2-dehydro-3-deoxyheptonate aldolase | VPBB_A1085 | 38.293 | 0.1225 | 0.0262 | aromatic amino acid family biosynthetic process |
| Q87S46 | Uncharacterized protein | VP0578 | 30.574 | 0.4831 | 0.0241 | polyphosphate kinase activity |
| Q87MJ3 | TyrA protein | VP2262 | 33.360 | 0.4487 | 0.0167 | heme binding,  peroxidase activity |
| E1D978 | 4-aminobutyrate transaminase | gabT | 45.135 | 0.1614 | 0.0218 | gamma-aminobutyric acid metabolic process |
| F3RWM9 | Formate--tetrahydrofolate ligase | fhs | 62.356 | 0.1995 | 0.0132 | folic acid-containing compound biosynthetic process |
| Q87KB5 | Threonine dehydratase | VP3062 | 56.603 | 0.0724 | 0.0020 | isoleucine biosynthetic process |
| L0HR83 | Autonomous glycyl radical cofactor | VP3062 | 13.929 | 0.0221 | 0.0070 | lyase activity |
| L0HVJ2 | GGDEF family member | VPBB_1392 | 36.931 | 0.3221 | 0.0152 | intracellular signal transduction |
| L0I1V6 | Uncharacterized protein | VPBB_2644 | 37.873 | 0.3873 | 0.0158 | carboxylesterase activity |
| L0I3I5 | Peptidase T | pepT | 44.775 | 0.1820 | 0.0005 | proteolysis |
| L0HZL6 | Thiazole biosynthesis protein ThiH | VPBB_2853 | 43.227 | 0.1888 | 0.0245 | thiamine biosynthetic process |
| L0HXQ6 | "Type cbb3 cytochrome oxidase biogenesis protein CcoG, involved in Cu oxidation | VPBB_2886 | 54.023 | 0.3698 | 0.0206 | electron carrier activity,  iron-sulfur cluster binding |
| Q87SL7 | DNA primase | VP0405 | 66.260 | 0.3908 | 0.0398 | DNA primase activity |
| E1DE29 | Sigma-54 dependent transcriptional regulator | VIPARAQ4037_0414 | 49.596 | 0.2089 | 0.0127 | regulation of transcription, DNA-dependent |
| Q9X9J8 | Polar flagellar FlgE homolog | flgE | 47.356 | 0.4207 | 0.0407 | ciliary or bacterial-type flagellar motility |
| Q87Q02 | "Peptidase, M20A family | VP1348 | 35.579 | 0.3698 | 0.0193 | hydrolase activity |
| E1EC13 | Triosephosphate isomerase | tpiA | 26.932 | 0.4055 | 0.0463 | glycolysis |
| Q87HN2 | "Molybdenum ABC transporter, periplasmic molybdenum-binding protein | VPA0931 | 27.039 | 0.0180 | 0.0011 | molybdate transmembrane-transporting ATPase activity |
| F3RXR1 | Putative alkaline phosphatase | VP10329_08537 | 46.785 | 0.0929 | 0.0037 | dephosphorylation |
| Q87GB3 | Uncharacterized protein | VPA1404 | 50.341 | 0.1786 | 0.0309 | integral to membrane |
| F3RTJ4 | Putative acetoin utilization protein AcuB | VP10329_12341 | 17.883 | 0.1225 | 0.0059 | metabolic process |
| Q87NY2 | Uncharacterized protein | VP1736 | 73.642 | 0.0319 | 0.0014 | sulfuric ester hydrolase activity |
| L0HZG6 | EAL family protein | VPBB_1817 | 92.560 | 0.0855 | 0.0153 | signal transducer activity |
| F3RP70 | Phosphoheptose isomerase | gmhA | 20.823 | 0.4831 | 0.0498 | lipopolysaccharide core region biosynthetic process |
| Q87P00 | Cytochrome c551 peroxidase | VP1718 | 38.315 | 0.1585 | 0.0259 | electron carrier activity |
| L0HX45 | Succinylglutamate desuccinylase | astE | 38.793 | 0.2168 | 0.0229 | arginine catabolic process to succinate |
| Q87FM1 | Uncharacterized protein | VPA1658 | 44.248 | 0.0406 | 0.0042 | metal ion binding |
| Q87LA1 | UTP-glucose-1-phosphate uridylyltransferase | VP2711 | 32.338 | 0.4246 | 0.0072 | biosynthetic process,  UDP-glucose metabolic process |
| Q87FT7 | Oxygen-insensitive NAD(P)H nitroreductase | VPA1591 | 24.463 | 0.0441 | 0.0022 | oxidation-reduction process |
| F3S1S8 | Putative outer membrane protein | VP10329_15175 | 22.969 | 0.0223 | 0.0007 | cell outer membrane |
| Q87P85 | Putative RTX toxin | VP1633 | 342.490 | 0.3981 | 0.0157 | calcium ion binding |
| L0HTX0 | Purine nucleoside phosphorylase | VPBB_0061 | 29.823 | 0.0809 | 0.0069 | nucleoside metabolic process |
| L0HY83 | Uracil phosphoribosyltransferase | upp | 22.887 | 0.3076 | 0.0265 | uracil salvage |
| L0I1K1 | Glutathione S-transferase | VPBB_A0591 | 24.275 | 0.4365 | 0.0450 | transferase activity |
| E1D4N7 | L-asparaginase 2 | VIPARAQ4037_A0341 | 38.003 | 0.0194 | 0.0267 | asparagine metabolic process |
| L0I3J7 | Transcriptional regulator VpsT family | VPBB_A0330 | 24.583 | 0.1432 | 0.0125 | transcription, DNA-dependent |
| Q87G70 | "Putative transcriptional regulator, LuxR family | VPA1447 | 25.406 | 0.1247 | 0.0465 | transcription, DNA-dependent |
| Q87LU4 | Carbonic anhydrase | VP2514 | 25.222 | 0.2312 | 0.0441 | carbon utilization |
| Q87KW5 | Superoxide dismutase | VP2860 | 22.988 | 0.2089 | 0.0434 | superoxide metabolic process |
| Q87NE1 | "Nitrite reductase, Fe-S protein (NrfC) | VP1927 | 24.936 | 0.0347 | 0.0139 | electron carrier activity,  iron-sulfur cluster binding |
| Q87GB2 | Putative polysaccharide export-related protein | VPA1405 | 19.863 | 0.0344 | 0.0112 | polysaccharide transmembrane transporter activity |
| Q87RD9 | Ferrous iron transport protein B | VP0858 | 82.556 | 0.4325 | 0.0428 | ferrous iron transmembrane transporter activity |
| L0I179 | "3,4-dihydroxy-2-butanone 4-phosphate synthase | ribB | 23.549 | 0.0296 | 0.0200 | riboflavin biosynthetic process |
| Q87IA0 | C4-dicarboxylate transporter, anaerobic | VPA0706 | 47.116 | 0.2421 | 0.0154 | C4-dicarboxylate transmembrane transporter activity |
| L0HVF7 | Anaerobic dimethylsulfoxide reductase chain B | VPBB_1359 | 23.167 | 0.0603 | 0.0019 | electron carrier activity,  iron-sulfur cluster binding |
| Q87R98 | Putative O-methyltransferase | VP0899 | 40.288 | 0.2512 | 0.0262 | O-methyltransferase activity |
| L0HSF2 | Cell division protein ZapB | zapB | 9.412 | 0.0847 | 0.0388 | barrier septum assembly,  cytokinesis by binary fission |
| Q87PV9 | Putative transcriptional regulator | VP1391 | 60.414 | 0.2535 | 0.0371 | regulation of transcription, DNA-dependent,  transcription, DNA-dependent |
| Q87NU3 | Putative oxidoreductase | VP1775 | 46.494 | 0.0347 | 0.0136 | oxidoreductase activity |
| A6AYQ6 | Anaerobic dimethyl sulfoxide reductase chain A | A79_6135 | 90.870 | 0.1148 | 0.0420 | electron carrier activity |
| Q87G72 | Putative secreted calcium-binding protein | VPA1445 | 19.789 | 0.0106 | 0.0201 | calcium ion binding |
| Q87K01 | Putative secretion protein | VPA0097 | 36.998 | 0.0187 | 0.0007 | transmembrane transport |
| Q87MM1 | RNA polymerase sigma factor | VP2210 | 22.461 | 0.0643 | 0.0178 | DNA-dependent transcription, initiation |
| F3RRR5 | Putative outer membrane porin protein | VP10329_21230 | 36.757 | 0.0213 | 0.0007 | no GO |
| E1D705 | PmbA protein | pmbA | 48.314 | 0.3908 | 0.0175 | no GO |
| Q847U9 | CpsH | cpsH | 33.655 | 0.0497 | 7.34 E-07 | no GO |
| L0I0Q2 | MSHA biogenesis protein MshN | VPBB_2522 | 39.088 | 0.2559 | 0.0103 | no GO |
| L0HX19 | UPF0304 protein VPBB_0942 | VPBB_0942 | 19.843 | 0.2421 | 0.0006 | no GO |
| Q87JN7 | Uncharacterized protein | VPA0211 | 32.082 | 0.1690 | 0.0447 | no GO |
| Q87SP8 | Uncharacterized protein | VP0374 | 26.965 | 0.4656 | 0.0397 | no GO |
| L0I1F8 | TldD protein | VPBB_2503 | 51.357 | 0.4831 | 0.0205 | no GO |
| F3RQU1 | Putative uncharacterized protein | VP10329_13495 | 17.881 | 0.4571 | 0.0437 | no GO |
| E1DA98 | UPF0319 protein VIPARAQ4037_2973 | VIPARAQ4037_2973 | 24.310 | 0.0904 | 0.0102 | no GO |
| Q87SY6 | Uncharacterized protein | VP0286 | 23.108 | 0.3873 | 0.0361 | no GO |
| F3RXI2 | Putative uncharacterized protein | VP10329_08322 | 29.487 | 0.1117 | 0.0436 | no GO |
| Q87Q28 | Putative LicD1 protein | VP1322 | 31.343 | 0.1330 | 0.0365 | no GO |
| F3RUA6 | Putative uncharacterized protein | VP10329_00565 | 83.403 | 0.1318 | 0.0427 | no GO |
| Q87KC6 | Uncharacterized protein | VP3051 | 10.360 | 0.2489 | 0.0400 | no GO |
| Q87R97 | Putative oxidoreductase protein | VP0900 | 45.988 | 0.1306 | 0.0070 | no GO |

a. Protein information including accession numbers, gene names, MW and Gene Ontology (GO) was taken from the UniProtKB

b. iTRAQ ratio of VPP compared with VPW

c. Statistical analysis of iTRAQ ratio of VPP compared with VPW was performed using unpaired t-test.
